# Supplementary material for: Reciprocal inhibition between TP63 and STAT1 regulates anti-tumor immune response through interferon-γ signaling in squamous cancer
Source: Nat Commun. 2024 Mar 20;15:2484. doi: 10.1038/s41467-024-46785-9 (PMC10954759; doi:10.1038/s41467-024-46785-9)
Supplement: Supplementary file 1 — Supplementary Information [file 41467_2024_46785_MOESM1_ESM.pdf]

Supplementary figures

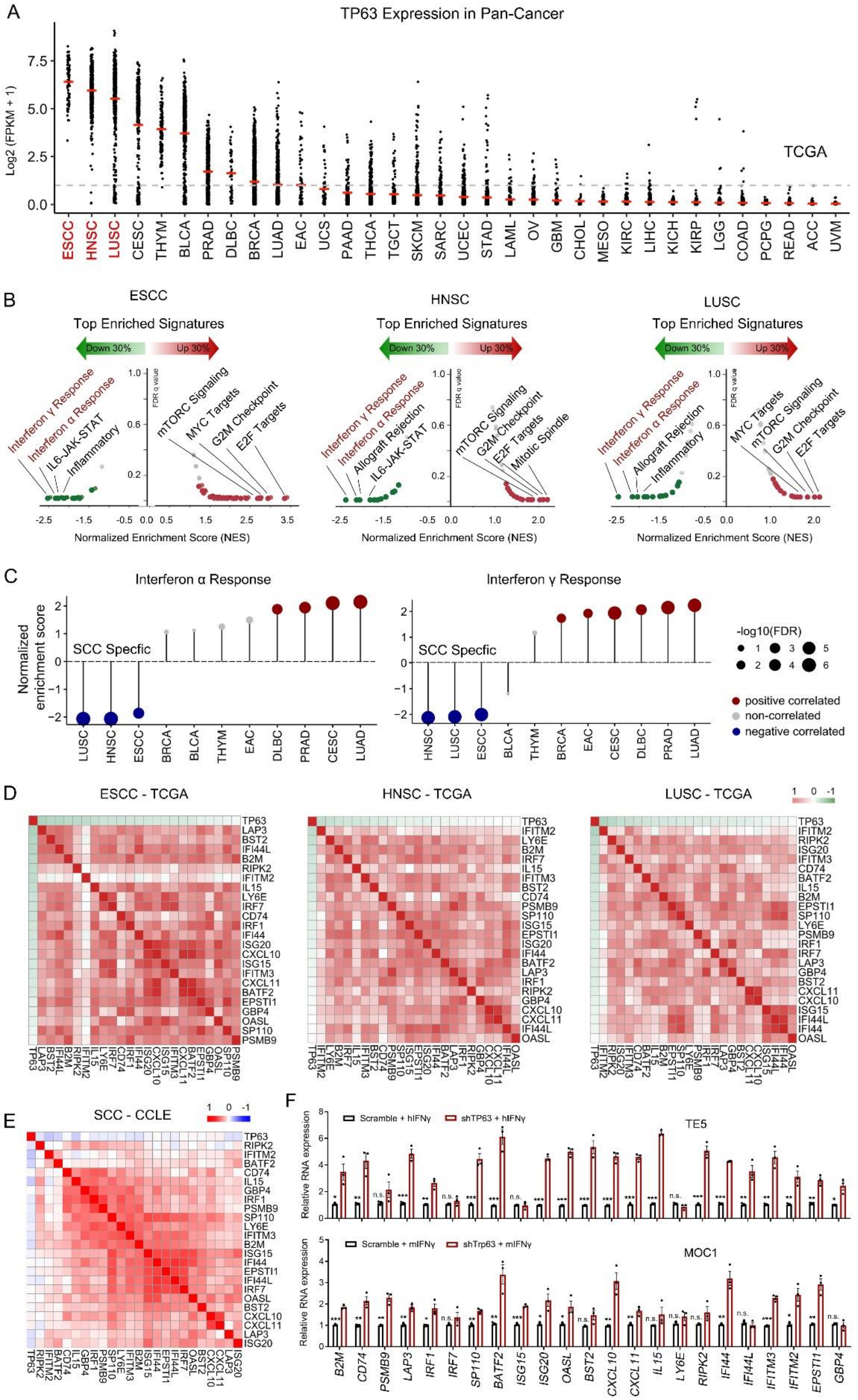

Supplementary Fig. 1. TP63 suppresses IFNγ response signaling and ISGs expression in SCCs, Related to Fig. 1. (A) The expression of *TP63* in pan-cancer primary tumors (TCGA), presented with $\log_2^{(FPKM+1)}$. Indications ranked by median *TP63* expression are denoted by red bars. **(B)** The top four enriched signatures that are negatively or positively correlated with the expression of *TP63* in three types of SCC tumors revealed by RNA-seq data and pathway enrichment analysis. RNA-seq data were from TCGA. **(C)** Cancer types that show negative or positive correlation between IFNγ/IFNα response and the expression of *TP63*. Cancer type with *TP63* average FPKM expression >=1 in TCGA project was chosen for Hallmark pathway enrichment analysis. **(D and E)** Pearson correlation matrix for 23 IFNγ/α response genes (ISGs) based on TCGA (**D**) and CCLE datasets (**E**). (B-D) n= 76 (ESCC), 500 (HNSC) and 501 (LUSC) independent patient samples, respectively. **(F)** qRT-PCR analysis showing relative mRNA levels of the 23 ISGs in TE5 and MOC1 cells expressing Scramble or sh*Trp63* pulsed with IFNγ (100 ng/mL) for 48 hours. Data represent mean ± SD, n=3 biologically independent experiments. The significance was determined by two-sided *t*-test. N.S., not significant $^*P < 0.05$, $^{**}P < 0.01$, $^{***} P < 0.001$. Source data and exact P values for 1F panel are provided as a Source Data file.

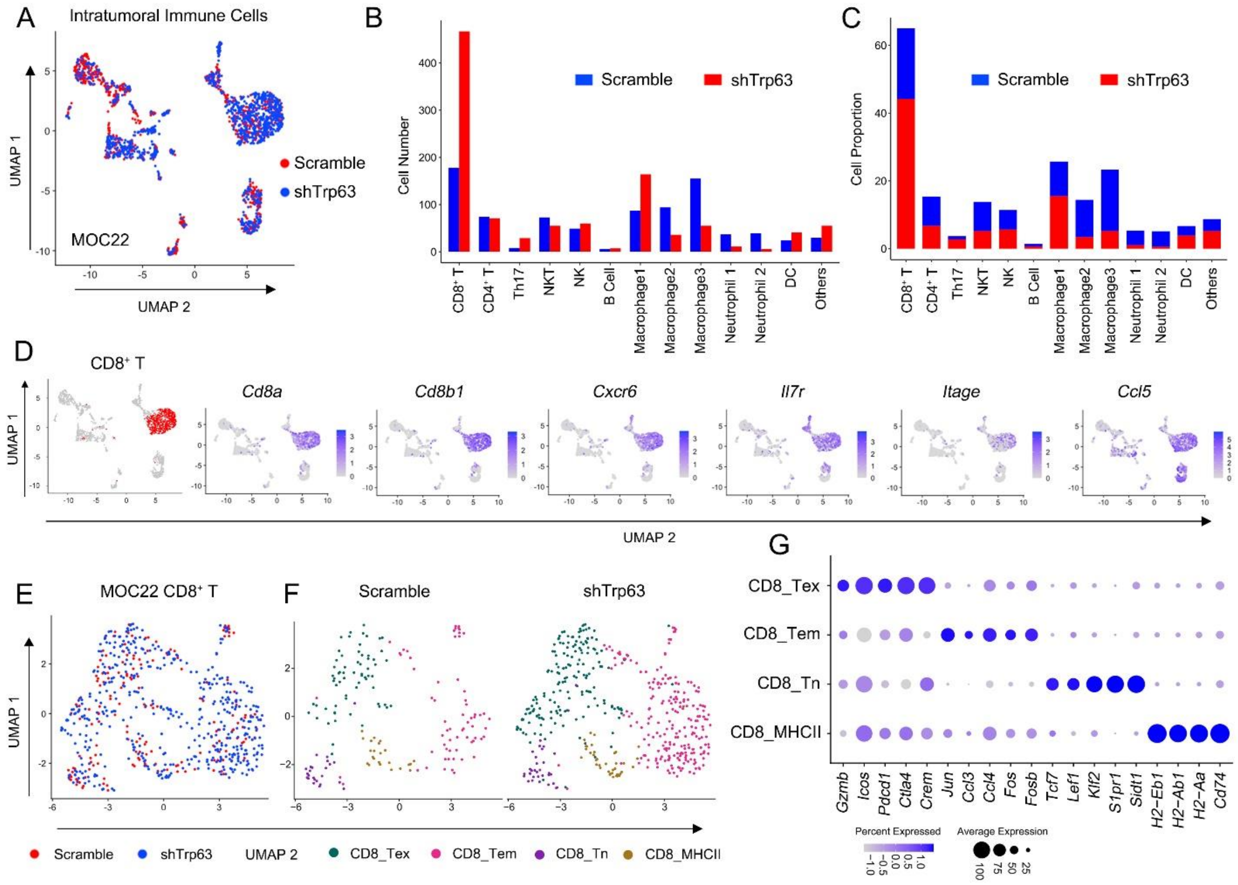

Supplementary Fig. 2. Downregulation of *TP63* promotes CD8$^+$ T cell infiltration and activation in murine SCC models, Related to Fig. 2. **(A)** UMAP plots displaying all the intratumoral immune cells in either Scramble (red) or sh*Trp63* (blue) MOC22 tumors. n = 1,911 cells. No significant batch effect was observed in different conditions of MOC22 tumors. **(B and C)** Bar plots showing the cell number (**B**) and cell proportion (**C**) of each group in Scramble and sh*Trp63* MOC22 tumors. 854 and 1,057 cells were analyzed in either Scramble and sh*Trp63* group. **(D)** UMAP plots showing the expression levels of representative mark genes in CD8$^+$ T cells. n = 1,911 cells. **(E and F)** UMAP visualization of 645 CD8$^+$ T cells from Scramble and sh*Trp63* tumors, colored by different conditions (**E**), or cell types in each condition (**F**). 178 cells in Scramble and 467 cells in sh*Trp63* group. **(G)** Dot plot showing the expression levels of representative marker genes across each CD8$^+$ T cell subgroup. n = 238 in CD8_Tex, 289 in CD8_Tem, 57 in CD8_Tn and 61 in CD8_MHCII subgroups.

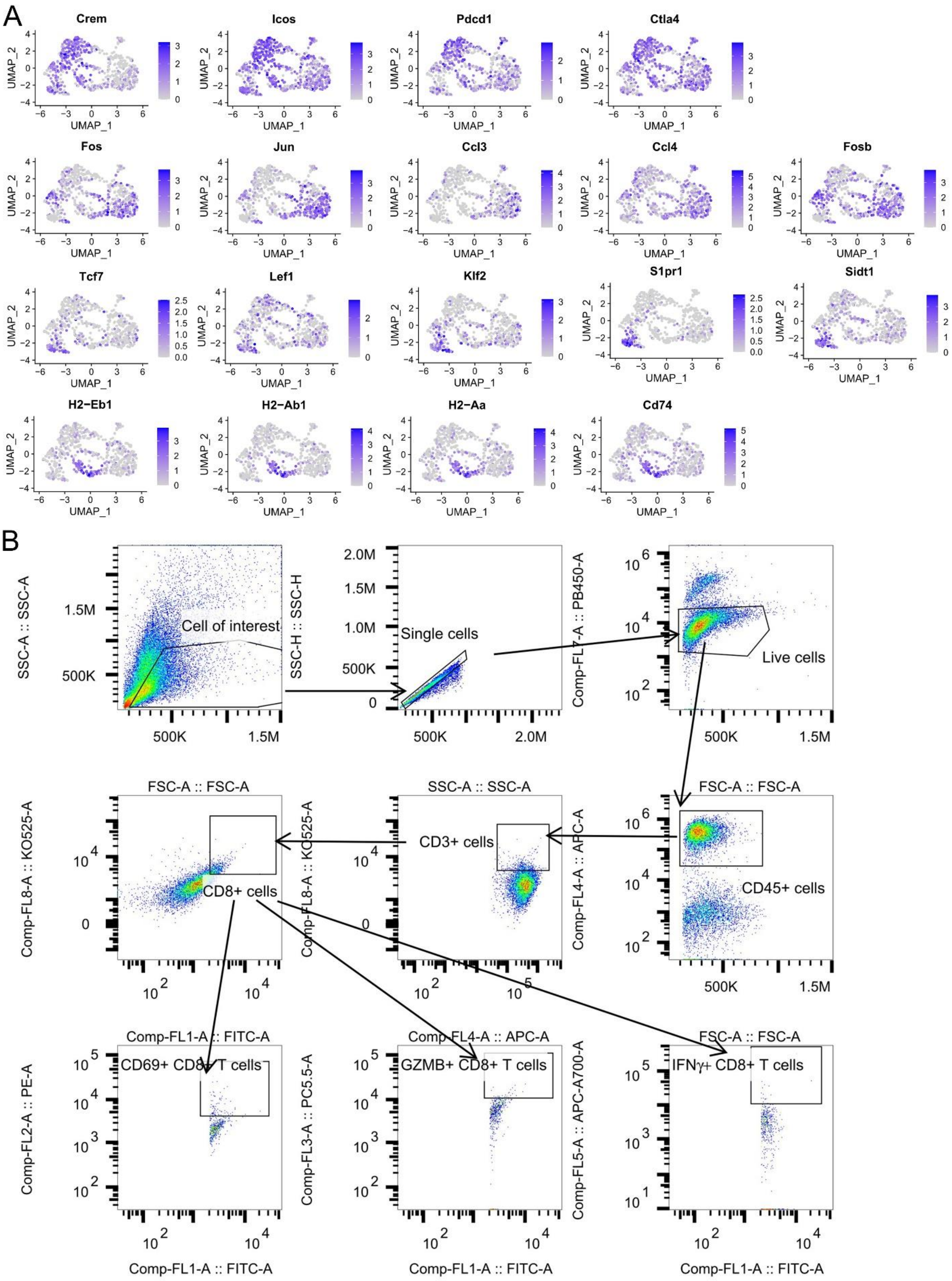
A
Crem
Icos
Pdcd1
Ctla4
Fos
Jun
Ccl3
Ccl4
Fosb
Tcf7
Lef1
Klf2
S1pr1
Sidt1
H2−Eb1
H2−Ab1
H2−Aa
Cd74
UMAP_1
UMAP_2
B
SSC-A :: SSC-A
Cell of interest
SSC-H :: SSC-H
Single cells
Comp-FL7-A :: PB450-A
Live cells
FSC-A :: FSC-A
Comp-FL8-A :: KO525-A
CD8+ cells
SSC-A :: SSC-A
CD3+ cells
Comp-FL4-A :: APC-A
CD45+ cells
Comp-FL1-A :: FITC-A
Comp-FL2-A :: PE-A
CD69+ CD8+ T cells
Comp-FL4-A :: APC-A
Comp-FL3-A :: PC5.5-A
GZMB+ CD8+ T cells
Comp-FL5-A :: APC-A700-A
IFNγ+ CD8+ T cells

Supplementary Fig. 3. Expression of TP63 changes CD8$^+$ T cell infiltration and activation of murine SCC tumors, Related to Fig. 2. (A) UMAP visualization of CD8$^+$ T cells with color-coded for the expression levels of representative mark genes. **(B)** The gating strategy and a representative staining example for immune profiling of murine SCC tumors. AKR and HNM007-allograft tumors were collected after 7 days induction of Doxycycline. Tumors were dissociated into single cell suspension; immune cells were enriched using CD45 MicroBeads. Single cells were first gated and followed by Fixable Viability Stain 450 selection for live cells. Immune cell populations were then identified by the sequential gating strategy using specific markers: total immune cells (CD45$^+$), total T cells (CD3$^+$), CD8$^+$ T cells. In addition, the expression of activation and cytotoxic protein markers (CD69, GZMB, IFNγ) in CD8$^+$ T cells was analyzed.

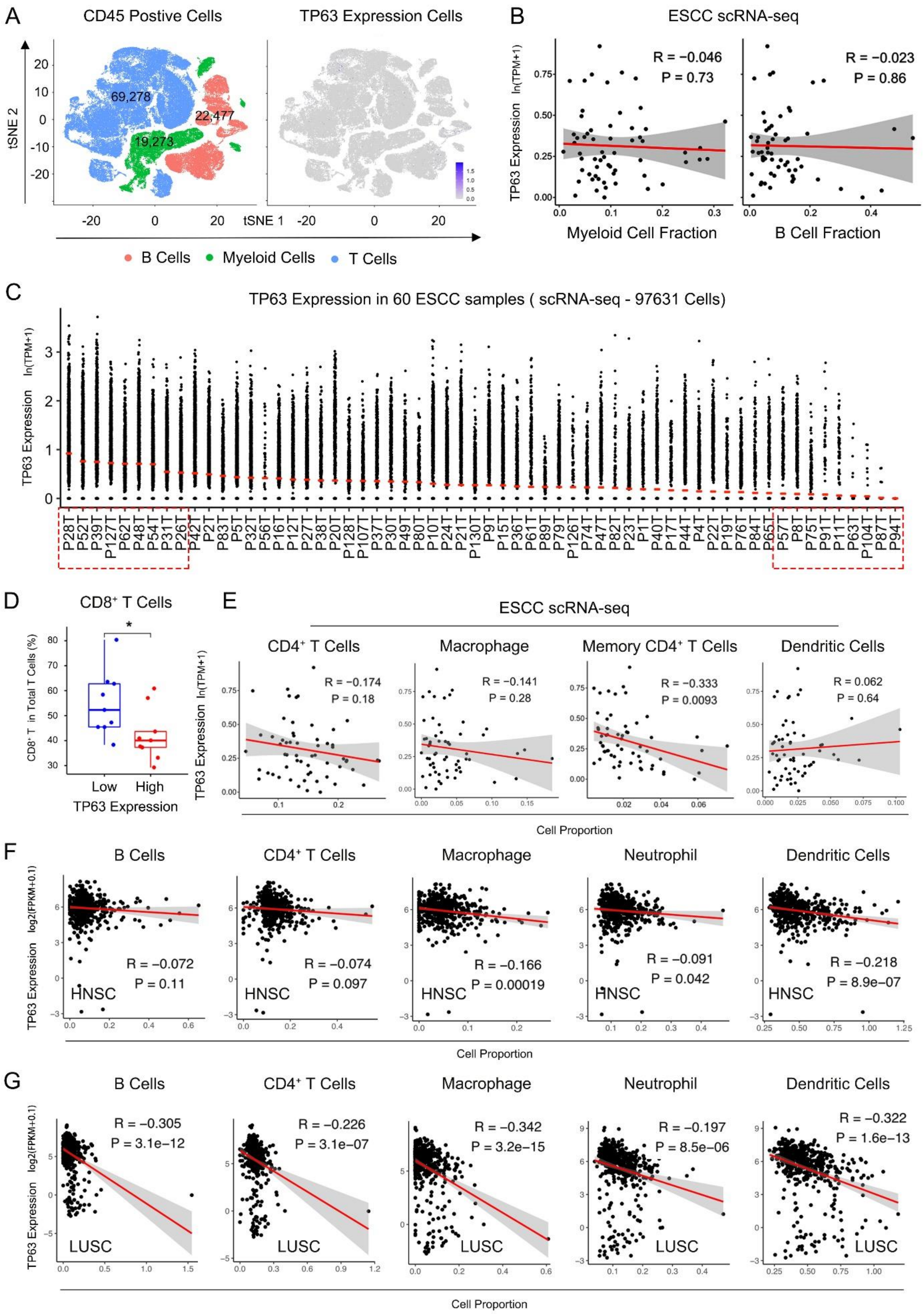
A
CD45 Postive Cells
TP63 Expression Cells
tSNE 2
tSNE 1
69,278
22,477
19,273
B Cells
Myeloid Cells
T Cells
B
ESCC scRNA-seq
R = −0.046
P = 0.73
R = −0.023
P = 0.86
TP63 Expression ln(TPM+1)
Myeloid Cell Fraction
B Cell Fraction
C
TP63 Expression in 60 ESCC samples (scRNA-seq - 97631 Cells)
TP63 Expression ln(TPM+1)
D
CD8+ T Cells
CD8+ T in Total T Cells (%)
Low
High
TP63 Expression
E
ESCC scRNA-seq
CD4+ T Cells
Macrophage
Memory CD4+ T Cells
Dendritic Cells
R = −0.174
P = 0.18
R = −0.141
P = 0.28
R = −0.333
P = 0.0093
R = 0.062
P = 0.64
TP63 Expression ln(TPM+1)
Cell Proportion
F
B Cells
CD4+ T Cells
Macrophage
Neutrophil
Dendritic Cells
R = −0.072
P = 0.11
R = −0.074
P = 0.097
R = −0.166
P = 0.00019
R = −0.091
P = 0.042
R = −0.218
P = 8.9e−07
HNSC
TP63 Expression log2(FPKM+0.1)
Cell Proportion
G
B Cells
CD4+ T Cells
Macrophage
Neutrophil
Dendritic Cells
R = −0.305
P = 3.1e−12
R = −0.226
P = 3.1e−07
R = −0.342
P = 3.2e−15
R = −0.197
P = 8.5e−06
R = −0.322
P = 1.6e−13
LUSC
TP63 Expression log2(FPKM+0.1)
Cell Proportion

Supplementary Fig. 4. Negative correlation between CD8$^+$ T cell infiltration and tumor-intrinsic TP63 expression in the TME of human ESCC, Related to Figs. 3 and 4. (A) t-SNE plots of 111,028 CD45$^+$ cells in 60 ESCC patient tumors and 4 adjacent normal tissue samples, colored by cell types (left) and *TP63* expression (right). The median expression of *TP63* in CD45$^+$ /CD45$^-$ cells in each ESCC patients was calculated. **(B)** Myeloid or B cell fractions showing no correlation with *TP63* expression. **(C)** Beeswarm plot showing *TP63* expression in each CD45$^-$ cell of each sample. Patient samples are ranked by the median expression level of *TP63* highlighted with red bars. The top/bottom 15% patient samples according to *TP63* expression are labeled with red dotted-line rectangle. *TP63*-low samples, n=9. *TP63*-high samples, n=9. **(D)** Box plots showing the cell proportion of CD8$^+$ T cells in total T cells of *TP63*-low/high expressing samples. The boxplot shows the median (central line), upper and lower quartiles (box limits), and min to max range (whiskers) analyzed by a two-sided *t*-test. P = 0.0361 (*). **(E)** Scatter plots revealing the correlation between different immune cell populations and the expression of *TP63* in ESCC patient tumors. scRNA-seq data of (**A-C**) were retrieved from GSE160269[1]. n = 60. **(F and G)** The correlation between different immune cell fractions and *TP63* expression in 500 HNSC (**F**) and 501 LUSC (**G**) patient samples. The immune cell fractions were predicted by TIMER2[2] using TCGA expression datasets. Pearson Correlation Coefficient was calculated in (**B** and **E-G**). The error bands show 95% confidence interval. R: Pearson's product-moment correlation; P value: Two-sided t-test.

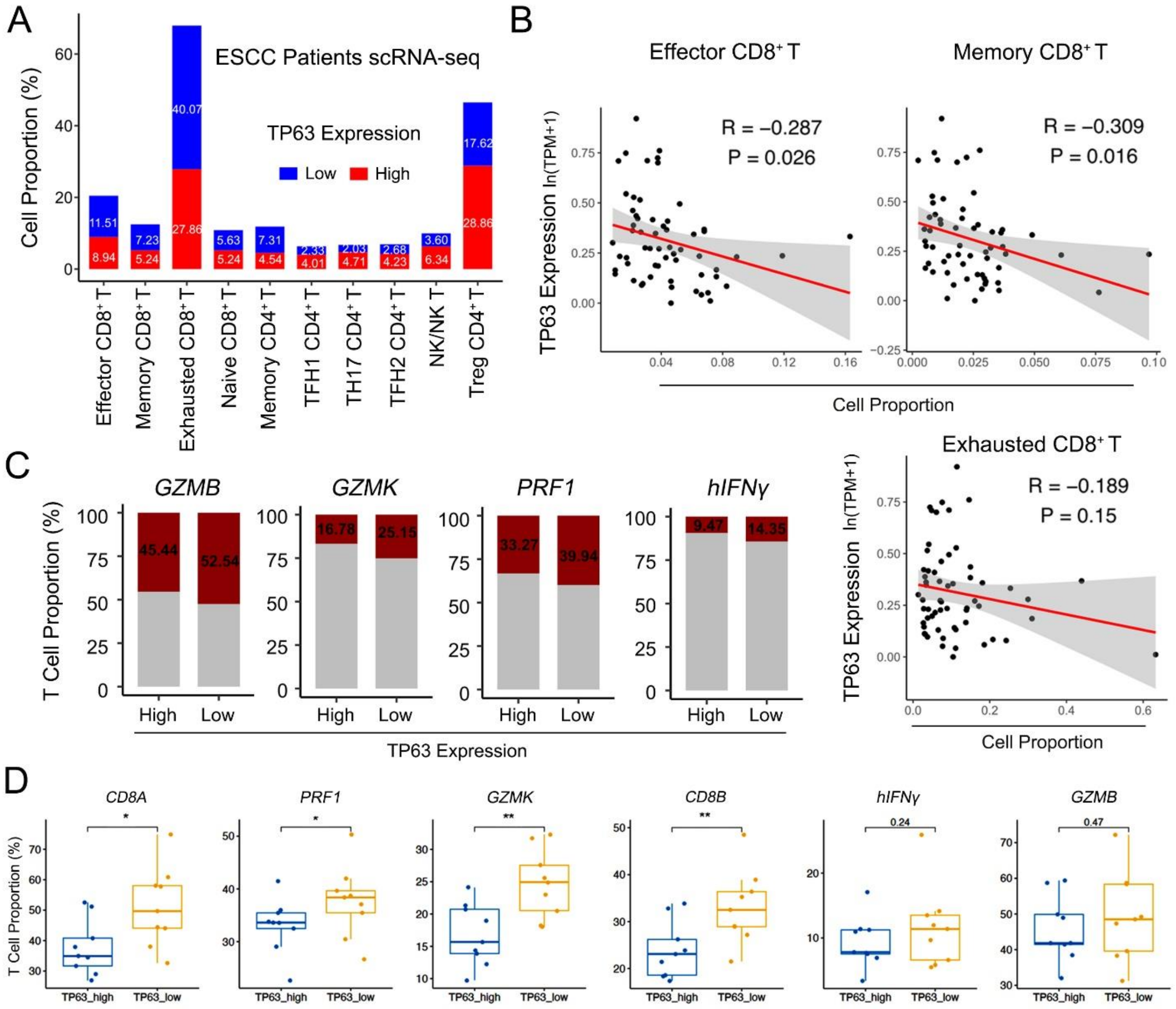

Supplementary Fig. 5. Over-expression of TP63 impairs CD8$^+$ T cell infiltration and activation, Related to Fig. 4. (A) Cell proportion of T cell subgroups in *TP63*-high/low expressing ESCC patient samples. n = 8,182 (effector CD8 T), 4,495 (Memory CD8 T), 21,893 (Exhausted CD8 T), 3,544 (Naïve CD8 T), 4,301 (Memory CD4 T), 2,578 (Tfh1 CD4 T), 2,504 (Th17 CD4 T), 1,811 (Tfh2 CD4 T), 3,839 (NK/NKT) and 16,131 (Treg CD4 T) cells in total. **(B)** The correlation between the abundance of effector/memory/exhausted CD8$^+$ T cells and the expression of *TP63* in ESCC patient tumors. n = 60. Pearson Correlation Coefficient was calculated. The error bands show 95% confidence interval. R: Pearson's product-moment correlation; P value: Two-sided t-test. **(C)** Bar plots showing the proportion of cytotoxic lymphocytes that express cytotoxic and activation markers including GZMB, GZMK, PRF1 and IFNγ in *TP63*-high/low expressing ESCC tumors. A total of 9,001 and 11,012 cells were analyzed in *TP63* high and *TP63* low group,

respectively. **(D)** Box plots showing the statistical analysis of (**C**) according to the cell proportion of T cells with the expression of indicated marker genes in *TP63*-low/high expressing patient tumor samples. n = 9 in each group. For each gene of interest, we defined target gene-positive cells if the corresponding TPM >=1. Subsequently, we calculated the fraction of target gene-positive T cells in *TP63*-low and *TP63*-high tumors. The boxplot shows the median (central line), upper and lower quartiles (box limits), and min to max range (whiskers). One-tailed Wilcox test was used to compared these two groups. P value in each group: 0.012 (*CD8A*), 0.047 (*PRF1*), 0.0053 (*GZMK*), 0.0071 (*CD8B*), 0.24 (*IFNG*), 0.47 (*GZMB*). *P < 0.05, **P < 0.01.

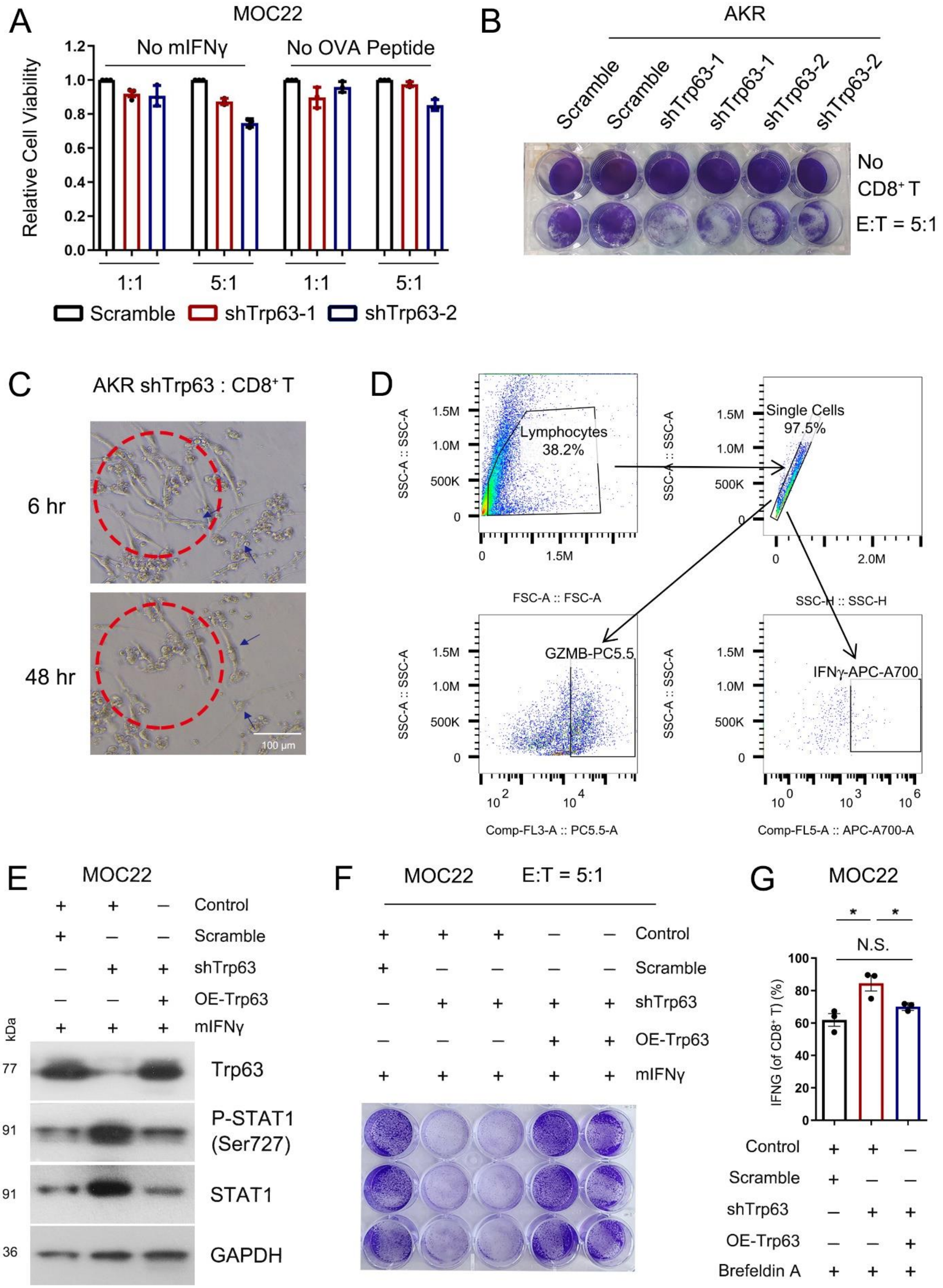
A
MOC22
No mIFNγ
No OVA Peptide
Relative Cell Viability
1:1
5:1
1:1
5:1
Scramble
shTrp63-1
shTrp63-2
B
AKR
Scramble
Scramble
shTrp63-1
shTrp63-1
shTrp63-2
shTrp63-2
No CD8⁺ T
E:T = 5:1
C
AKR shTrp63 : CD8⁺ T
6 hr
48 hr
100 μm
D
Lymphocytes 38.2%
Single Cells 97.5%
GZMB-PC5.5
IFNγ-APC-A700
SSC-A :: SSC-A
FSC-A :: FSC-A
SSC-H :: SSC-H
Comp-FL3-A :: PC5.5-A
Comp-FL5-A :: APC-A700-A
E
MOC22
Control
Scramble
shTrp63
OE-Trp63
mIFNγ
kDa
77
91
91
36
Trp63
P-STAT1 (Ser727)
STAT1
GAPDH
F
MOC22
E:T = 5:1
Control
Scramble
shTrp63
OE-Trp63
mIFNγ
G
MOC22
N.S.
IFNG (of CD8⁺ T) (%)
Control
Scramble
shTrp63
OE-Trp63
Brefeldin A

Supplementary Fig. 6. TP63 suppresses IFNγ/STAT1 signaling and CD8^{+} T-cell killing, Related to Fig. 5. (A) Relative cell viability of Scramble and sh*Trp63* MOC22 cells pulsed with or without IFNγ/OVA peptide in co-culture with OT-I CD8^{+} T cells at indicated effector: target (E: T) ratios. The results were repeated in three biologically independent experiments. **(B)** Crystal violet staining of Scramble and sh*Trp63* AKR cells incubation with or without OT-I CD8^{+} T cells at the indicated effector: target (E: T) ratios. **(C)** Bright field images showing representative sh*Trp63* AKR cells that were killed by activated OT-I CD8^{+}T cells following 48 hr co-culture. Red circles highlight lost AKR cells; Blue arrows indicate the location of cells. Murine Scramble/sh*Trp63* SCC cells were pretreated with IFNγ (10 ng/mL) for 24 hr before pulsing with OVA peptide in (B) and (C). **(D)** The exemplification of the gating strategy for intracellular cytokine stains in *ex vivo* co-culturing system (related to Figures 5E and 5F). After 48 hr of co-culturing SCC and CD8 T cells, adherent living cancer cells were stained crystal violet, while suspended CD8 T cells were collected for flow cytometry analysis of GZMB and IFNγ. Single cells were first gated. The expression of activation and cytotoxic protein markers (GZMB, IFNγ) in the co-culturing system was analyzed. The results of (**B-D**) were repeated with three biologically independent experiments in two cell lines. **(E)** Western blotting analysis showing the protein levels of TP63, p-STAT1 and STAT1 in the indicated conditions in MOC22 cells. MOC22 cells were transfected with siRNA targeting *Trp63* or non-targetable control (Scramble) and ectopically expressed TP63 or empty vector (Control) in the presence of IFNγ (100 ng/mL). *Trp63*-KD: *Trp63* knockdown; OE-*Trp63*: ectopic expression of *Trp63*. **(F)** Crystal violet staining of MOC22 cells pretreated with IFNγ (10 ng/mL) and incubation with OT-I CD8+ T cells for 24 hr at 5:1 effector: target (E: T) ratio. The results of (**E** and **F**) were repeated in two biologically independent experiments. **(G)** Percent of IFNγ^{+} production in Scramble, sh*Trp63* and *TP63*-overexpressing MOC22 and OT-I T cell co-cultures. Brefeldin A was added for the last 4 hr before harvest of cells. Data represent mean ± SD, n=3 biologically independent experiments. The significance was determined by two-sided *t*-test. P value: 0.0213 (*Trp63*-KD *vs.* Scramble), 0.0442 (*Trp63*-KD *vs.* *Trp63*-KD + OE-*Trp63*), 0.1160 (Scramble *vs.* *Trp63*-KD + OE-*Trp63*) *P < 0.05; N.S., not significant. Source data are provided as a Source Data file.

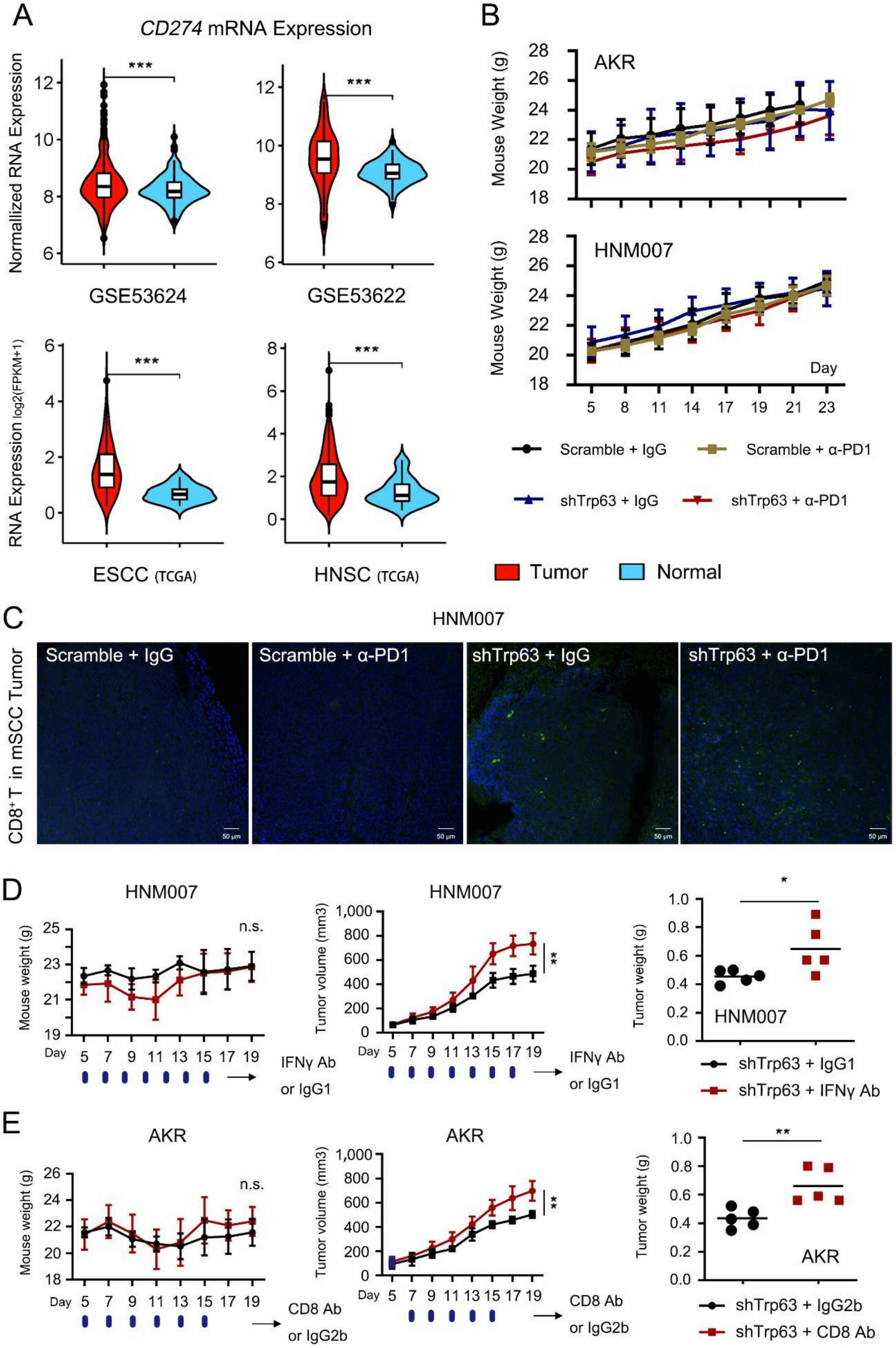
A
CD274 mRNA Expression
Normalized RNA Expression
***
GSE53624
GSE53622
RNA Expression log2(FPKM+1)
ESCC (TCGA)
HNSC (TCGA)
B
AKR
HNM007
Mouse Weight (g)
Day
5 8 11 14 17 19 21 23
Scramble + IgG
Scramble + α-PD1
shTrp63 + IgG
shTrp63 + α-PD1
Tumor
Normal
C
HNM007
CD8⁺ T in mSCC Tumor
Scramble + IgG
Scramble + α-PD1
shTrp63 + IgG
shTrp63 + α-PD1
50 μm
D
HNM007
Mouse weight (g)
n.s.
Day 5 7 9 11 13 15 17 19
IFNγ Ab
or IgG1
Tumor volume (mm3)
**
Tumor weight (g)
*
HNM007
shTrp63 + IgG1
shTrp63 + IFNγ Ab
E
AKR
Mouse weight (g)
n.s.
CD8 Ab
or IgG2b
Tumor volume (mm3)
**
Tumor weight (g)
**
AKR
shTrp63 + IgG2b
shTrp63 + CD8 Ab

Supplementary Fig. 7. Tumor reduction in the syngeneic mouse tumor model by downregulation of *Trp63* is CD8^{+} T cell-dependent, Related to Fig. 5. (A) Relative mRNA levels of *CD274* (PD-L1) in different cohorts of SCC samples. Data were retrieved from GSE53624, GSE53622 and TCGA database. Statistical significance were calculated using a two-tailed *t* test. n and P values in each group: 119 T (tumor) *vs.* 119 N (normal)/1.8E-04 (GSE53624), 60 T *vs.* 60 N/3.8E-04 (GSE53622), 76 T *vs.* 9 N/4.6E-04 (ESCC from TCGA), 500 T *vs.* 44 N/4.9E-05 (HNSC from TCGA). ***P < 0.001. **(B)** Body weight curve of SCC tumor-bearing mice received PD-1 mAb treatment or IgG isotype control (IgG2a). n=5 for each group. **(C)** Representative IF staining results showing the CD8^{+} T cells abundance in Scramble and sh*Trp63* HNM007 allografts, tumor-bearing mice treated with either PD-1 mAb or IgG isotype control (Scale bar, 50 μm). The similar results were observed in three biologically independent samples. **(D)** Body weights, tumor growth curves and tumor weights at completion of the study of *shTrp63* HNM007-bearing mice treated with IFNγ blocking antibody (n=5) or IgG isotype control (IgG1) (n=5). mAbs were given by intraperitoneal injection (i.p., 400 μg/injection/mouse) once every 2 days for up to 2 weeks. The significance was determined by a one-sided *t* -test. For the comparison between sh*Trp63* + IgG1 and sh*Trp63* + IFNγ Ab group: P = 0.9416 for mouse weight, P = 0.001 for tumor volume and P = 0.0405 for tumor weight. **(E)** Body weights, tumor growth curves and tumor weights at completion of the study of sh*Trp63* AKR-bearing mice treated with CD8 mAb (CD8 T-cell-depletion antibody) (n=5) or IgG isotype control (IgG2b) (n=5). mAbs were given by intraperitoneal injection (i.p., 100 μg/injection/mouse) once every 2 days for up to 2 weeks. The significance was determined by a one-sided *t* -test. For the comparison between sh*Trp63* + IgG1 and sh*Trp63* + IFNγ Ab group: P = 0.2441 for mouse weight, P = 0.001 for tumor volume and P = 0.0072 for tumor weight. N.S., not significant; *P < 0.05, **p < 0.01.

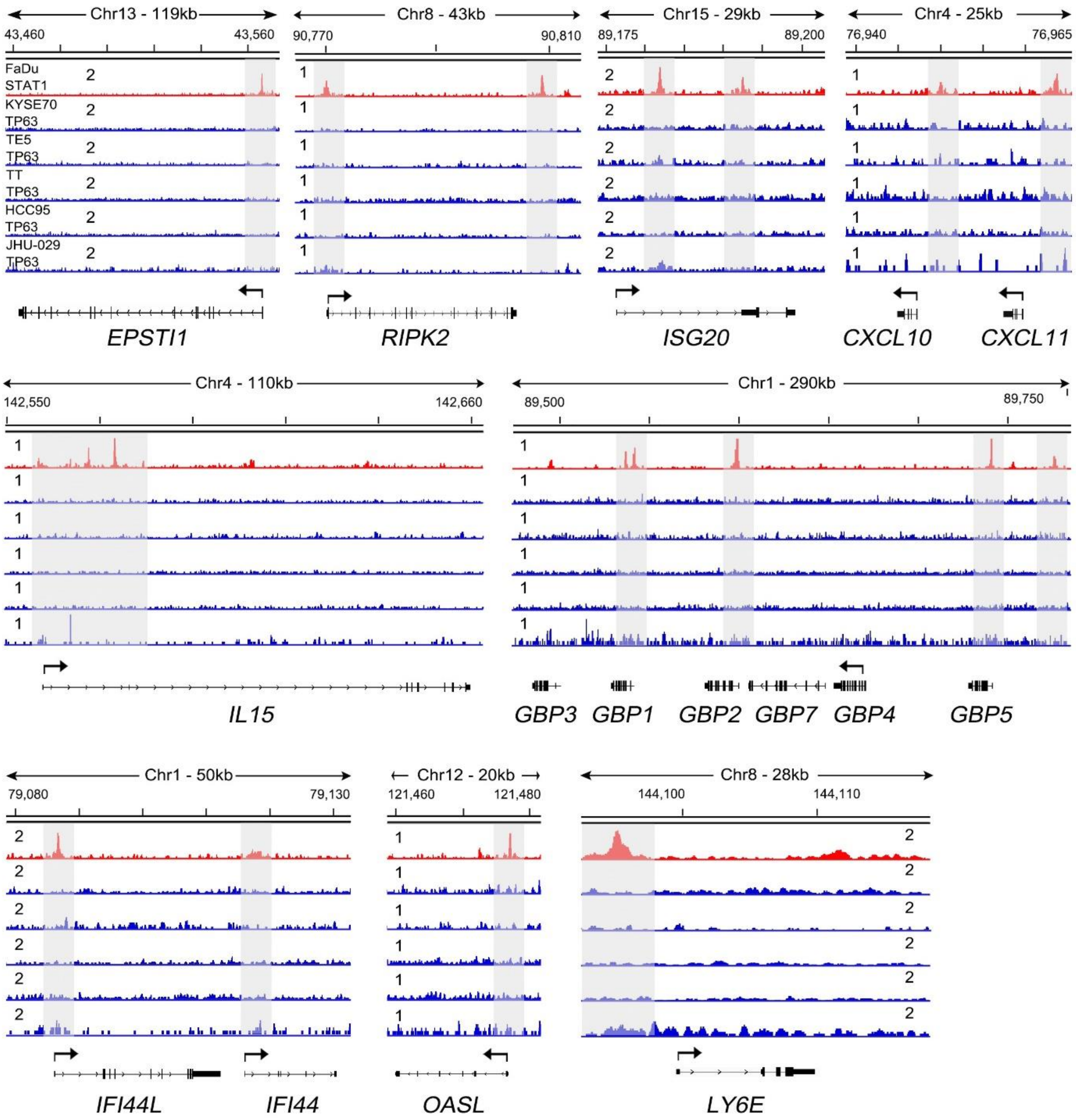

Supplementary Fig. 8. Occupancy of STAT1 but not TP63 at ISGs, Related to Fig. 6. IGV tracks of ChIP-seq revealing binding peaks for STAT1 (red) and TP63 (blue) on the promoter or enhancer loci of indicated IFN response genes. Grey shadows highlighting promoter region of each gene.

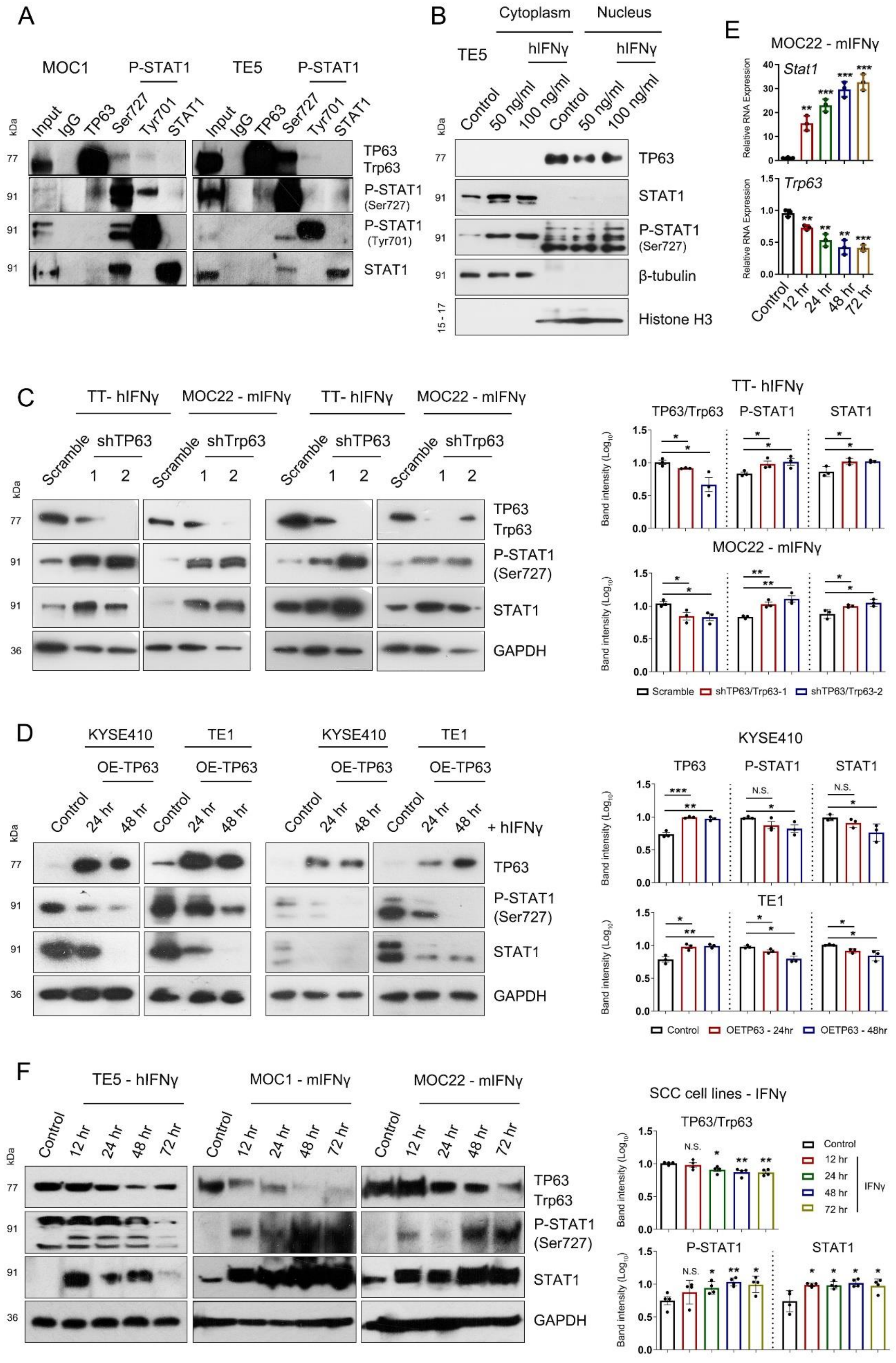
A
MOC1
P-STAT1
TE5
Input
IgG
TP63
Ser727
Tyr701
STAT1
TP63
Trp63
P-STAT1 (Ser727)
P-STAT1 (Tyr701)
B
Cytoplasm
Nucleus
TE5
hIFNγ
Control
50 ng/ml
100 ng/ml
β-tubulin
Histone H3
E
MOC22 - mIFNγ
Stat1
Trp63
Relative RNA Expression
12 hr
24 hr
48 hr
72 hr
C
TT- hIFNγ
MOC22 - mIFNγ
Scramble
shTP63
shTrp63
GAPDH
TP63/Trp63
Band intensity (Log10)
shTP63/Trp63-1
shTP63/Trp63-2
D
KYSE410
TE1
OE-TP63
+ hIFNγ
Control
OETP63 - 24hr
OETP63 - 48hr
N.S.
F
TE5 - hIFNγ
MOC1 - mIFNγ
SCC cell lines - IFNγ
IFNγ
kDa

Supplementary Fig. 9. Reciprocal inhibition between TP63 and IFNγ-STAT1 signaling, Related to Fig. 6. (A) Co-IP followed Western blotting analysis showing the protein interaction between TP63 and phosphorylated STAT1 (Ser727 and Tyr701) in both murine MOC1 and human TE5 cells. **(B)** Western blotting analysis revealing the subcellular localization of TP63 and p-STAT1 (Ser727) at cytoplasm and nucleus of TE5 cells stimulated with IFNγ at different concentrations. The results were repeated in two biologically independent experiments. **(C-F)** Independent biological or/and experimental replicates (related to Figures 6F, 6G and 6I) of western blotting analysis (left) and statistical densitometry analyses (right) of left panel showing levels of TP63, STAT1 and p-STAT1 in the indicated cells and conditions, including knockdown *TP63* (**C**), overexpression of *TP63* (**D**) and a time-dependent change of mRNA (**E**) and protein levels (**F**). IFNγ (100 ng/mL) was administrated for 48 hr in (**C**). *TP63*-KD: *TP63* knockdown; OE-*Trp63*: ectopic over-expression of *TP63*. The results of panel (**A** and **F**) were repeated in four biologically independent cell lines. The results of (**C**-**E**) were repeated with three biologically independent experiments in two cell lines. Bars of (**C-F**) represent mean ± SD. The significance was determined by two-sided *t*-test. P value for the comparison of TP63, p-STAT1 and STAT1 protein level between Scramble and sh*TP63*-1/-2 in (**C**): 0.0437/0.0363, 0.0491/0.0367, 0.0419/00261 in TT cells; 0.0384/0.0258, 0.0059/0.0049, 0.0445/0.0284 in MOC22 cells. P value for the comparison of TP63, p-STAT1 and STAT1 protein level between Control and OE-TP63-24hr/-48 in (**D**): 9.82E-04/0.0025, 0.1260/0.0483, 0.1740/0.0468 in KYSE410 cells; 0.0148/0.0076, 0.0434/0.0111, 0.0105/0.0235 in TE1 cells. P value for the comparison of *STAT1* and *TP63* mRNA level between Control and IFNγ treatment for 12 hr/24 hr/48 hr/72 hr in (**E**): 0.0012/1.24E-04/9.60E-05/8.07E-05 for *STAT1*; 0.0038/0.0028/0.0018/1.91E-04 for *Trp63*. P value for the comparison of TP63, p-STAT1 and STAT1 protein level between Control and IFNγ treatment for 12 hr/24 hr/48 hr/72 hr in (**F**): 0.5180/0.0123/0.0052/0.0018 for TP63; 0.2851/0.0465/0.0064/0.0315 for p-STAT1; 0.0230/0.0300/0.0175/0.0489 for STAT1. N.S., not significant; *P < 0.05, **P < 0.01, ***P < 0.001. Source data are provided as a Source Data file.

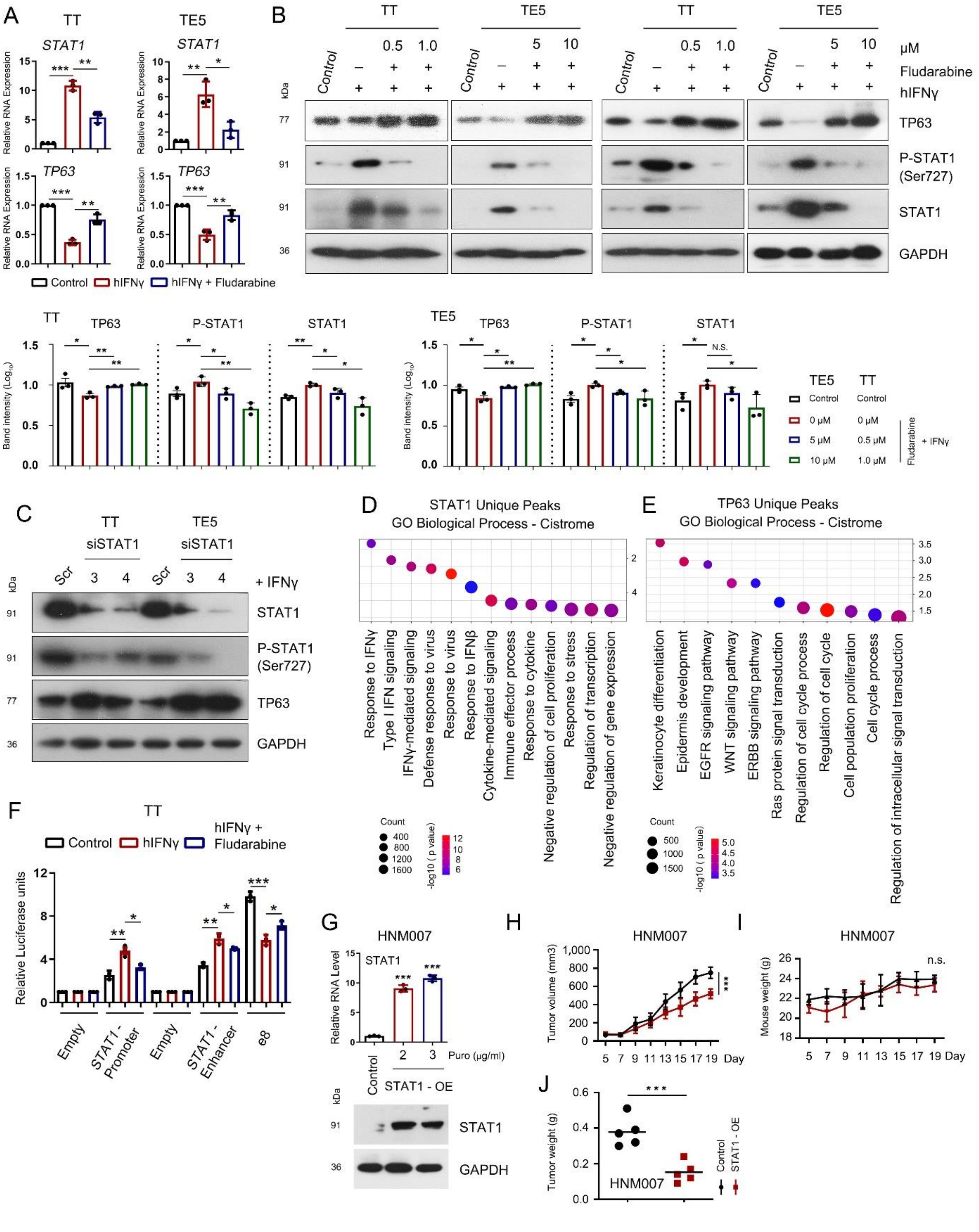

Supplementary Fig. 10. Reciprocal transcriptional inhibition between TP63 and STAT1, Related to Fig. 7. (A) qRT-PCR analysis revealing mRNA expression of *STAT1* and *TP63* in

both TT and TE5 cells treated with IFNγ (100 ng/mL) +/− Fludarabine (1 μM and 10 μM for TT and TE5 respectively), a STAT1-specific inhibitor. P value for the comparison of Control *vs.* IFNγ and IFNγ *vs.* IFNγ + Fludarabine group: 3.04E-05/0.0016 for *STAT1*, 2.14E-05/0.0023 for *TP63* in TT cells; 0.0032/0.0154 for *STAT1*, 0.0006/0.0088 for *TP63* in TE5 cells. **(B)** Two independent experimental replicates (related to Figure 6J) of western blotting analysis (upper) and statistical densitometry analyses (lower) of upper panel showing levels of TP63, STAT1 and p-STAT1 in both TT and TE5 cells treated with IFNγ (100 ng/mL) or/and Fludarabine, a STAT1-specific inhibitor. P value for the comparison of Control *vs.* IFNγ treatment, IFNγ *vs.* IFNγ + Fludarabine (0.5 µM /1 µM in TT or 5 µM/10 µM in TE5): 0.0492/0.0058/0.0033 for TP63, 0.0465/0.0452/0.0034 for p-STAT1, 0.0014/0.05/0.0130 for STAT1 in TT cells; 0.0485/0.0128/0.0060 for TP63, 0.0195/0.0116/0.0396 for p-STAT1, 0.0396/0.107/0.0441 for STAT1 in TE5 cells. **(C)** Western blotting assays showing protein levels of indicated proteins upon knockdown of STAT1 (with #3 and #4 siRNA target) or not. The results were repeated in three biologically independent experiments. **(D and E)** Functional categories of STAT1- (D) or TP63- (E) uniquely occupied regions in SCC cells. **(F)** Relative luciferase activity of pGL3-enhancer (1st Empty), pGL3-enhancer+STAT1 promoter, pGL3-promoter (2nd Empty), pGL3-promoter+ STAT1 enhancer, pGL3-promoter+e8 upon stimulation with IFNγ (100 ng/mL) +/− Fludarabine (1 µM) in TT cells. Relative luciferase activity comparison of Control *vs.* IFNγ, IFNγ *vs.* IFNγ + Fludarabine: P = 0.0041 and P = 0.0101 for *STAT1*-promoter group, P = 0.0013 and P = 0.032 for *STAT1*-enhancer group, P = 0.0004 and P = 0.0201 for e8 group. (**G**) qRT-PCR (upper) and western blotting (lower) analysis showing mRNA and protein levels of STAT1 in STAT1-overexpressing HNM007 cells. Stably *STAT1*-overexpressed cells were selected with puromycin at the concentration either 2 µg/mL or 3 µg/mL. Puro: puromycin. P value for the comparison of Control *vs.* Puro (2 µg/mL or 3 µg/mL) selection group: 1.61E-05/4.94E-06. **(H-J)** Tumor growth curves **(H)**, Body weights **(I)** and Tumor weights at completion of the study **(J)** of HNM007-bearing mice with either STAT1-overexpression (n=5) or empty vector control (n=5). (**A, B, F** and **G**) Data represent mean ± SD, n=3 biologically independent experiments. P values were determined using a two-sided *t* -test. The significance of (**H-J**) was determined by a one-sided *t*-test. P value for each group: 0.0002 (**H**), 0.1332 (**I**), 9.97E-04 (**J**). N.S., not significant; *P < 0.05, **P < 0.01, ***P < 0.001. Source data are provided as a Source Data file.

Supplementary Table 1. Key Resources Used in This Study.

REAGENT or RESOURCE	SOURCE	IDENTIFIER
**Antibodies**		
Rabbit Anti-p63 Antibody	Abcam	Cat# ab97865; 1:1000 for WB; 1:200 for IF
Rabbit Anti-p63-α Antibody	Cell Signaling Technology	Cat# 13109; clone D2K8X; 1:1000 for WB; 1:500 for IF; 1:100 for IP and ChIP
Rabbit Anti-p63 Antibody	GeneTex	Cat# GTX102425; clone N2C1; 1:1000 for WB; 1:400 for IF
Mouse Anti-CD8α	Cell Signaling Technology	Cat# 70306; clone C8/144B; 1:300 for IF
CD8 Monoclonal Antibody	Thermo Fisher Scientific	Cat# MA5-14548; clone SP16; 1:300 for IF
APC anti-mouse CD45 Antibody	BioLegend	Cat# 103112; clone 30-F11
Brilliant Violet 510™ anti-mouse CD3 Antibody	BioLegend	Cat# 100233; clone 17A2
FITC anti-mouse CD8a Antibody	BioLegend	Cat# 100706; clone 53-6.7
PE anti-mouse CD69 Antibody	BioLegend	Cat# 104507; clone H1.2F3
PerCP/Cyanine5.5 anti-human Granzyme B Recombinant	BioLegend	Cat# 372212; clone QA16A02
Alexa Fluor® 700 anti-mouse IFN-γ Antibody	BioLegend	Cat# 505823; clone XMG1.2
Purified Rat Anti-Mouse CD16/CD32 (Mouse BD Fc Block™)	BD Biosciences	Cat# 553142;<br>Clone 2.4G2 (RUO)
InVivoMab anti-mouse PD-1	BioXcell	Cat# BE0146; clone RMP1-14
InVivoMAb rat IgG2a isotype control	BioXcell	Cat# BE0089; clone 2A3
InVivoMab anti-mouse CD8α	BioXcell	Cat# BE0061; clone 2.43
InVivoMAb rat IgG2b isotype control	BioXcell	Cat# BE0090; clone LTF-2
InVivoMAb anti-mouse IFNγ	BioXcell	Cat# BE0055; clone XMG1.2
InVivoMAb rat IgG1 isotype control	BioXcell	Cat# BE0088; clone HRPN
Phospho-STAT1 (Ser727) Polyclonal Antibody	Invitrogen	Cat# 44-382G; 1: 500 for WB; 1:200 for IF; 1: 50 for IP
STAT1 [p Tyr701] Antibody	Novus Biologicals	Cat# AF2894-SP; 1:1000 for WB; 1: 50 for IP;
Stat1 Antibody	Cell Signaling Technology	Cat# 9172; 1:1000 for WB; 1: 50 for IP
Rabbit Anti-GAPDH (14C10)	Cell Signaling Technology	Cat# 2118; 1:2000 for WB
Peroxidase AffiniPure Goat Anti-Rabbit IgG (H+L)	Jackson ImmunoResearch Laboratories, Inc	Cat# 111-035-045; 1:10000 for WB

Peroxidase AffiniPure Goat Anti-Mouse IgG (H+L)	Jackson ImmunoResearch Laboratories, Inc	Cat# 115-035-003; 1:10000 for WB
Mouse anti-Human BATF2 / SARI Antibody	LSBio	Cat# LS-C541060; 1:1000 for WB
Beta-2-Microglobulin Polyclonal antibody	Proteintech	Cat# 13511-1-AP; 1:4000 for WB
PD-L1/CD274 Monoclonal antibody	Proteintech	Cat# 66248-1-Ig; 1:2000 for WB
IFNGR1 Rabbit Polyclonal Antibody	Novus Biologicals	Cat# AF7176; 1:500 for WB
IFNGR2 Polyclonal antibody	Proteintech	Cat# 10266-1-AP; 1:1000 for WB
IRF1 Polyclonal antibody	Proteintech	Cat# 11335-1-AP; 1:500 for WB
MHC class I (HLA-A/B) Rabbit mAb	ABclonal	Cat# A8754; 1:1000 for WB
Rabbit secondary antibody	Abcepta	Cat# ASP1615; 1:10000 for WB
Mouse secondary antibody	Abcepta	Cat# ASP1613; 1:10000 for WB
Fixable Viability Stain 450	BD Biosciences	Cat# 562247
Donkey anti-Mouse IgG (H+L) Highly Cross-Adsorbed Secondary Antibody, Alexa Fluor™ 488	Invitrogen	Cat# A-21202; 1:5000 for IF
Donkey anti-Mouse IgG (H+L) Highly Cross-Adsorbed Secondary Antibody, Alexa Fluor™ 594	Invitrogen	Cat# A-21203; 1:1000 for IF
Donkey anti-Rabbit IgG (H+L) Highly Cross-Adsorbed Secondary Antibody, Alexa Fluor™ 488	Invitrogen	Cat# A-21206; 1:1000 for IF
Donkey anti-Rabbit IgG (H+L) Highly Cross-Adsorbed Secondary Antibody, Alexa Fluor™ 594	Invitrogen	Cat# A-21207; 1:3000 for IF
Rabbit secondary antibody	GE Healthcare	Cat# NXA934; 1:10000 for WB
Mouse secondary antibody	GE Healthcare	Cat# NXA931; 1:10000 for WB
**Bacterial and Virus Strains**		
TStbl3 Chemically Competent Cell	Tsingke Biotechnology	Cat# TSC-C06
TOP10 Chemically Competent Cell	Tsingke Biotechnology	Cat# TSC-C12
**Biological Samples**		
Human ESCC slides	This study	N/A
SCC allograft slides	This study	N/A

**Chemicals, Peptides, and Recombinant Proteins**		
jetPRIME® Transfection Reagent	Polyplus	Cat# 101000046
Lipofectamine™ 2000 Transfection Reagent	Thermo Fisher Scientific	Cat# 11668019
Lipofectamine™ RNAiMAX Transfection Reagent	Thermo Fisher Scientific	Cat# 13778150
Polybrene	Sigma-Aldrich	Cat# H9268
Puromycin	Sigma-Aldrich	Cat# 540222
Doxycycline	Sigma-Aldrich	Cat# 324385
Recombinant mouse Interferon gamma protein	Abcam	Cat# ab9922
IFN-gamma human	Sigma-Aldrich	Cat# SRP3058
Fludarabine	MecChemExpress	Cat# HY-B0069
IMDM	Hyclone	Cat# sh30228.02
Ham's Nutrient Mixture F12	Hyclone	Cat# sh30026.01
Fetal Bovine Serum, FBS	Omega Scientific	Cat# FB-02
Fetal Bovine Serum, FBS	Wisent	Cat# 086-150
Insulin	Sigma-Aldrich	Cat# I6634
Insulin	absin	Cat# abs9169
Hydrocortisone	Sigma-Aldrich	Cat# H0135
Recombinant Human EGF	Peprotech	Cat# AF-100-15
Recombinant Murine IL-2	Peprotech	Cat# 212-12
SIINFEKL ($OVA_{257-264}$)	Sigma-Aldrich	Cat# s7951
Collagenase, Type IV	Invitrogen	Cat# 17104019
DNase I	Roche	Cat# 10104159001
0.5% Trypsin-EDTA	Thermo Fisher Scientific	Cat# 15400054
Isoflurane	RWD	Cat# R510-22-10
Dynabeads™ Protein G for Immunoprecipitation	Thermo Fisher Scientific	Cat# 10004D
BeyoMag™ Protein A+G	Beyotime	Cat# P2108
**Critical Commercial Assays**		
RNeasy Mini Kit	QIAGEN	Cat# 74106
Maxima™ H Minus cDNA Synthesis Master Mix with dsDNase	Thermo Fisher Scientific	Cat# M1682
PowerUp™ SYBR™ Green Master Mix	Thermo Fisher Scientific	Cat# A25918
Steady Pure Universal RNA Extraction Kit	Accurate Biology	Cat# AG21017
Evo M-MLV RT Premix	Accurate Biology	Cat# AG11707
SYBR® Green Premix Pro Taq HS qPCR Kit	Accurate Biology	Cat# AG11701
Pro Taq HS PCR	Accurate Biology	Cat# AG11307
ZymoPURE II Plasmid Kits	Zymo Research	Cat# D4200
TIANpure Mini Plasmid Kit	TIANGEN	Cat# DP104-02
Pierce™ Rapid Gold BCA Protein Assay Kit	Thermo Fisher Scientific	Cat# A53225

MojoSort™ Mouse CD8 T Cell Isolation Kit	Biolegend	Cat# 480007
RBC Lysis Buffer	Biolegend	Cat# 420301
Tumor Dissociation Kit, mouse	Miltenyi Biotec	Cat# 130-096-730
CD45 (TIL) MicroBeads, mouse	Miltenyi Biotec	Cat# 130-110-618
True-Nuclear™ Transcription Factor Buffer Set	BioLegend	Cat# 424401
Dual-Luciferase® Reporter Assay System	Promega	Cat# E1960
Dual-Luciferase® Reporter Assay System	YEASEN	Cat# 11402ES60
**Experimental Models: Cell Lines**		
TE5<br>TT	Dr. Koji Kono<br>(Cancer Science Institute of Singapore, Singapore)	N/A
AKR<br>HNM007	Dr Anil K. Rustgi<br>(Columbia University Irving Medical Center, USA)	N/A
MOC1<br>MOC22	The laboratory of Ravindra Uppaluri (Dana-Farber Cancer Institute, USA)	N/A
**Experimental Models: Organisms/Strains**		
C57BL/6J	The Jackson Laboratory	Strain# 000664<br>RRID:IMSR_JAX:000664
C57BL/6-Tg (TcraTcrb)1100Mjb/J	The Jackson Laboratory	Strain# 003831<br>RRID:IMSR_JAX:003831
C57BL/6JGpt	Gem Pharmatech	Cat# N000013
C57BL/6-Tg (TcraTcrb)1100Mjb/J	Dr. Zhengfan Jiang<br>Peking University, China	N/A
**Oligonucleotides**		
qRT-PCR primers, see Table S2	Integrated DNA Technologies; Tsingke Biotechnology	N/A
shRNA sequences, see Table S2	Integrated DNA Technologies; Tsingke Biotechnology	N/A
**Recombinant DNA**		
pLKO.1-TRC	pLKO.1 - TRC cloning vector was a gift from David Root	Addgene plasmid #10878; RRID:Addgene_10878
pLKO.1-Scramble, pLKO.1-sh*TP63*-1, pLKO.1-sh*TP63*-2	Jiang et al.	N/A
Tet-pLKO-puro	Tet-pLKO-puro was a gift from Dmitri Wiederschain	Addgene plasmid #21915; RRID:Addgene_21915
Tet-pLKO-Scramble	This study	N/A

Tet-pLKO-sh*Trp63*-1	This study	N/A
Tet-pLKO-sh*Trp63*-2	This study	N/A
psPAX2	psPAX2 was a gift from Didier Trono	Addgene plasmid # 12260; RRID:Addgene_12260
pMD2.G	pMD2.G was a gift from Didier Trono	Addgene plasmid # 12259; RRID:Addgene_12259
deltaNp63alpha-FLAG	deltaNp63alpha-FLAG was a gift from David Sidransky	Addgene plasmid # 26979; RRID: Addgene_26979
pCDH-CMV-MCS-EF1-CopGFP-T2A-Puro	MIAOLING BIOLOGY	Cat# P29673
pCDH-CMV-STAT1(mouse)-3×FLAG-EF1a-Puro	MIAOLING BIOLOGY	Cat# P0268
**Software and Algorithms**		
GraphPad Prism 8.0	GraphPad Software	http://www.graphpad.com/
Cell Ranger v7.0.0	Zheng et al.[3]	https://support.10xgenomics.com/single-cell-gene-expression/software/pipelines/latest/what-is-cell-ranger
Seurat v.4.1.0	Stuart et al.[4]	https://github.com/satijalab/seurat
SAMtools v1.7	Li et al.[5]	https://github.com/samtools/samtools
MarkDuplicates v.1.136	N/A	https://broadinstitute.github.io/picard/
MACS2 v2.2.6	Liu et al.[6]	http://github.com/taoliu/MACS
HOMER	Heinz et al.[7]	http://homer.ucsd.edu/homer/index.html
FlowJo v.10.8	FlowJo, LLC	RRID: SCR_008520
**Deposited Data**		
scRNA-seq MOC22 tumors	This study	GSE221938 [https://www.ncbi.nlm.nih.gov/geo/query/acc.cgi?acc=GSE221938]
scRNA-seq data of human ESCC	Zhang et al.[1]	GSE160269 [https://www.ncbi.nlm.nih.gov/geo/query/acc.cgi?acc=GSE160269]
RNA Expression Data	TCGA	www.cbioportal.org
RNA Expression Data	CCLE	https://portals.broadinstitute.org/ccle
RNA-seq data of TE5	Jiang et al.[8]	GSE106564 [https://www.ncbi.nlm.nih.gov/geo/query/acc.cgi?acc=GSE106564]
RNA-seq data of FaDu	Saladi et al.[9]	GSE88833 [https://www.ncbi.nlm.nih.go

		v/geo/query/acc.cgi?acc=GSE88833]
RNA-seq data of SCC-6	Barbieri et al.[10]	GSE4975 [https://www.ncbi.nlm.nih.gov/geo/query/acc.cgi?acc=GSE4975]
STAT1 ChIP-seq	Ao et al.	GSE78212 [https://www.ncbi.nlm.nih.gov/geo/query/acc.cgi?acc=GSE78212]
TP63 ChIP-seq<br>H3K27ac ChIP-seq	Watanabe et al.[11]<br><br><br><br>Jiang et al.[8]<br><br><br><br>Jiang et al.[12]	GSE46837 [https://www.ncbi.nlm.nih.gov/geo/query/acc.cgi?acc=GSE46837]<br>GSE106563 [https://www.ncbi.nlm.nih.gov/geo/query/acc.cgi?acc=GSE106563]<br>GSE148920 [https://www.ncbi.nlm.nih.gov/geo/query/acc.cgi?acc=GSE148920]
**Other**		
RIPA Lysis Buffer	Merck Millipore	Cat# 20-188
RIPA buffer	Solarbio	Cat# R0020
cOmplete™, EDTA-free Protease Inhibitor Cocktail Tablets	Roche	Cat# 4693132001
Phosphatase Inhibitor Cocktail Tablets	Roche	Cat# 04906837001
PageRuler™ Prestained Protein Ladder	Thermo Fisher Scientific	Cat# 26616
ColorMixed Protein Marker	Solarbio	Cat# PR1920
Immobilon-P PVDF Membrane	Merck Millipore	Cat# IPVH00010
Amersham ECL Western Blotting Detection Reagent	GE Healthcare Life Sciences	Cat# RPN2106
Mounting medium with DAPI	ZSGB-BIO	Cat# ZLI-9557
ECL Chemiluminescence Kit	Vazyme	Cat# E412-02

WB: Western Blotting; IF: Immunofluorescence; IP: Immunoprecipitation.

Supplementary Table 2. Oligonucleotides used for siRNA, shRNA, quantitative real-time PCR and DNA fragment amplification.

	Forward	Reverse
si*Trp63*-1	GAGCAUGUCACCGAGGUUGUGAAAC	GUUUCACAACCUCGGUGACAUGCUCAG
si*Trp63*-2	GGCACUGAAUUCACAACAGUCCUGT	ACAGGACUGUUGUGAAUUCAGUGCCAA

si*Trp63*-3	CAAGAAAGCUGAGCAUGUCACCGA G	CUCGGUGACAUGCUCAGCUUUCU UGUA
si*Trp63*-4	GUCAUCUGAUUCGAGUAGAAGGG AA	UUCCCUUCUACUCGAAUCAGAUG ACUG
sh*Trp63*-1	CCGGGGCACTGAATTCACAACAGT CCTGTCTCGAGACAGGACTGTTGTG AATTCAGTGCCTTTTTG	AATTCAAAAAGGCACTGAATTCA CAACAGTCCTGTCTCGAGACAGG ACTGTTGTGAATTCAGTGCC
sh*Trp63*- 2	CCGGGTCATCTGATTCGAGTAGAA GGGAACTCGAGTTCCCTTCTACTCG AATCAGATGACTTTTTG	AATTCAAAAAGTCATCTGATTCGA GTAGAAGGGAACTCGAGTTCCCTT CTACTCGAATCAGATGAC
B2M	CGCTACTCTCTCTTTCTGGC	CAGACACATAGCAATTCAGG
CD74	CCAGCGCGACCTTATCTCC	TCACCAGGATGGAAAAGCCTG
PSMB9	TCCATGGGATAGAACTGGAGGA	ATGGCAAAAGGCTGTCGAGT
LAP3	CAATGCCGCCACCTTAACAG	TGCTGGCCTCGAAGAGTTTG
IRF1	ATGCTTCCACCTCTCACCAA	CATGTAGCCTGGAACTGTGT
IRF7	GAGCTGTGCTGGCGAGAAG	GGAGTCCAGCATGTGTGTGT
SP110	CCTGCGTGAATATCCCAATC	CAGGGTGAACAGCTTGGTTG
BATF2	CACCCTCAGACCCCTCCTAA	GAACTTCCACCCGGTCATGG
ISG15	AGGCAGCGAACTCATCTTTG	AGCTCTGACACCGACATGGA
ISG20	GGCTACACAATCTACGACAC	CTCGGATTCTCTGGGAGATT
OASL	ATTGTGCCTGCCTACAGAGC	ATGTCTCGTGCCCTCTGCT
BST2	AGGAGCTTGAGGGAGAGATCA	ACTTCTTGTCCGCGATTCTCA
CXCL10	CCTGCAAGCCAATTTTGTCCA	TGATGGCCTTCGATTCTGGAT
CXCL11	CAAATCGAAGCAAGCAAGGC	TCAGATGCTCTTTTCCAGGACTT
IL15	TTTCAGTGCAGGGCTTCCTAA	GGGTGAACATCACTTTCCGTAT
LY6E	TTGGTTTGTGACCTCCAGGC	TCACGAGATTCCCAATGCCG
RIPK2	AAACTTCAAGGTCCCTGCCA	GATCACAGAATGCAGCCCTT
IFI44	CGATGCGAAGATTCACTGGA	TCGTATTTGTTGAACCAGGG
IFI44L	CCATCTCTGAAGGACAGGAT	CGGCTTTGAGAAGTCATAGA
IFITM3	TACTCCGTGAAGTCTAGGGA	CAGTGATGCCTCCTGATCTA
IFITM2	GCCCTGATTTTGGGCATCTTCA	GATGCCTCCTGATCTATCGCT
EPSTI1	ACCCGCAATAGAGTGGTGAA	TGTATGCACTTGTGCGCCT
GBP4	CGGCTGGACTTTCAGCTTCTA	TCTGGATAACCTGGTGTGGG
STAT1	ACAGCAGAGCGCCTGTATTG	CTGCAGACTCTCCGCAACTA
GAPDH	GAAGGTGAAGGTCGGAGTC	GAAGATGGTGATGGGATTTC
B2m	TACGCCTGCAGAGTTAAGCA	GATCACATGTCTCGATCCCAGT
Cd74	CCTTGCTGATGCGTCCAATG	CTGGGTCATGTTGCCGTACT
Psmb9	GGGACAACCATCATGGCAGT	CAGCAGCGGAACCTGAGAG
Lap3	TGACGAAGGGCCTTGTTTTAG	AGAGGAGGTCCAGATATGTTCAA
Irf1	CAGCCGAGACACTAAGAGCAA	GCTGCTGAGTCCATCAGAGA
Irf7	GCGTACCCTGGAAGCATTTC	GCACAGCGGAAGTTGGTCT
Sp110	ATGAAGGTGAACATCGCCTATG	GGACAGAGGGACCAGATTTTG
Batf2	AGAAGCAGAAGAACCGAGTGG	AAGGATTCGTGCTGCTGGTG
Isg15	GGTGTCCGTGACTAACTCCAT	TGGAAAGGGTAAGACCGTCCT
Isg20	GACCCGAGGGAGAGATCAC	CAGGGCATTGAAGTCGTGCT
Oasl1	TGCTCAAGGTACTCAAGGTAGG	TGGGTACTCTGTTAGTCACACTC
Bst2	TGTAGAGACGGGTTGCGAG	CAGGGACTCCTGAAGGGTC
Cxcl10	CCAAGTGCTGCCGTCATTTTC	GGCTCGCAGGGATGATTTCAA
Cxcl11	TGTAATTTACCCGAGTAACGGC	CACCTTTGTCGTTTATGAGCCTT

Il15	CATCCATCTCGTGCTACTTGTG	GCCTCTGTTTTAGGGAGACCT
LY6E	GGGCATGGAGCAAGTTCATTC	GGCCACAGGCAGTTTATATTGTT
Ripk2	ATGCCACCTGAGAACTATGAGC	GCAAAGGATTGGTGACCTCTT
Ifi44	AACTGACTGCTCGCAATAATGT	GTAACACAGCAATGCCTCTTGT
Ifi44l	TCAGTTCAACCCCTGTGAGC	TGGACATTCTGAACCTGGCTT
Ifitm3	CCCCCAAACTACGAAAGAATCA	ACCATCTTCCGATCCCTAGAC
Ifitm2	TGGGCTTCGTTGCCTATGC	AGAATGGGGTGTTCTTTGTGC
Epsti1	TTGCAGCAAAACCGGAGACA	TCTCGTTTGGTGCTATCAGGG
Gbp4	GGAGAAGCTAACGAAGGAACAA	TTCCACAAGGGAATCACCATTTT
Stat1	TTCCCTCCTGGGCCTGATTA	AGATCCCGTACAGATGTCCA
Gapdh	CATCACTGCCACCCAGAAGACTG	ATGCCAGTGAGCTTCCCGTTCAG
STAT1-Promoter	TTCTCTATCGATAGGTAGCAGGAAAGCGAAACTACCC	GATCGCAGATCTCGAGGCCGCGTCTAATTGGCTGAG
STAT1-Enhancer	TTCTCTATCGATAGGTAAGCACTATCACATTCGCGGT	GATCGCAGATCTCGAGGTTTCCGAAGCGGTTCAAACT

Supplementary References

1 Zhang, X. *et al.* Dissecting esophageal squamous-cell carcinoma ecosystem by single-cell transcriptomic analysis. *Nat Commun* **12**, 5291, doi:10.1038/s41467-021-25539-x (2021).

2 Li, T. *et al.* TIMER: A Web Server for Comprehensive Analysis of Tumor-Infiltrating Immune Cells. *Cancer Res* **77**, e108-e110, doi:10.1158/0008-5472.CAN-17-0307 (2017).

3 Zheng, G. X. *et al.* Massively parallel digital transcriptional profiling of single cells. *Nat Commun* **8**, 14049, doi:10.1038/ncomms14049 (2017).

4 Stuart, T. *et al.* Comprehensive Integration of Single-Cell Data. *Cell* **177**, 1888-1902 e1821, doi:10.1016/j.cell.2019.05.031 (2019).

5 Li, H. *et al.* The Sequence Alignment/Map format and SAMtools. *Bioinformatics* **25**, 2078-2079, doi:10.1093/bioinformatics/btp352 (2009).

6 Liu, T. Use model-based Analysis of ChIP-Seq (MACS) to analyze short reads generated by sequencing protein-DNA interactions in embryonic stem cells. *Methods Mol Biol* **1150**, 81-95, doi:10.1007/978-1-4939-0512-6_4 (2014).

7 Heinz, S. *et al.* Simple combinations of lineage-determining transcription factors prime cis-regulatory elements required for macrophage and B cell identities. *Mol Cell* **38**, 576-589, doi:10.1016/j.molcel.2010.05.004 (2010).

8 Jiang, Y. *et al.* Co-activation of super-enhancer-driven CCAT1 by TP63 and SOX2 promotes squamous cancer progression. *Nat Commun* **9**, 3619, doi:10.1038/s41467-018-06081-9 (2018).

9 Saladi, S. V. *et al.* ACTL6A Is Co-Amplified with p63 in Squamous Cell Carcinoma to Drive YAP Activation, Regenerative Proliferation, and Poor Prognosis. *Cancer Cell* **31**, 35-49, doi:10.1016/j.ccell.2016.12.001 (2017).

10 Barbieri, C. E., Tang, L. J., Brown, K. A. & Pietenpol, J. A. Loss of p63 leads to increased cell migration and up-regulation of genes involved in invasion and metastasis. *Cancer Res* **66**, 7589-7597, doi:10.1158/0008-5472.CAN-06-2020 (2006).

11 Watanabe, H. *et al.* SOX2 and p63 colocalize at genetic loci in squamous cell carcinomas. *J Clin Invest* **124**, 1636-1645, doi:10.1172/JCI71545 (2014).

12 Jiang, Y. Y. *et al.* TP63, SOX2, and KLF5 Establish a Core Regulatory Circuitry That Controls Epigenetic and Transcription Patterns in Esophageal Squamous Cell Carcinoma Cell Lines. *Gastroenterology* **159**, 1311-1327 e1319, doi:10.1053/j.gastro.2020.06.050 (2020).
