# Supplementary material for: Reciprocal inhibition between TP63 and STAT1 regulates anti-tumor immune response through interferon-γ signaling in squamous cancer
Source: Nat Commun. 2024 Mar 20;15:2484. doi: 10.1038/s41467-024-46785-9 (PMC10954759; doi:10.1038/s41467-024-46785-9)
Supplement: Supplementary file 3 — Reporting Summary [file 41467_2024_46785_MOESM3_ESM.pdf]

nature portfolio

Corresponding author(s): Yan-Yi Jiang

Last updated by author(s): Mar 2, 2024

Reporting Summary

Nature Portfolio wishes to improve the reproducibility of the work that we publish. This form provides structure for consistency and transparency in reporting. For further information on Nature Portfolio policies, see our Editorial Policies and the Editorial Policy Checklist.

Statistics

For all statistical analyses, confirm that the following items are present in the figure legend, table legend, main text, or Methods section.

n/a	Confirmed	
☐	☒	The exact sample size (n) for each experimental group/condition, given as a discrete number and unit of measurement
☐	☒	A statement on whether measurements were taken from distinct samples or whether the same sample was measured repeatedly
☐	☒	The statistical test(s) used AND whether they are one- or two-sided<br>*Only common tests should be described solely by name; describe more complex techniques in the Methods section.*
☒	☐	A description of all covariates tested
☐	☒	A description of any assumptions or corrections, such as tests of normality and adjustment for multiple comparisons
☐	☒	A full description of the statistical parameters including central tendency (e.g. means) or other basic estimates (e.g. regression coefficient) AND variation (e.g. standard deviation) or associated estimates of uncertainty (e.g. confidence intervals)
☐	☒	For null hypothesis testing, the test statistic (e.g. F, t, r) with confidence intervals, effect sizes, degrees of freedom and P value noted<br>*Give P values as exact values whenever suitable.*
☒	☐	For Bayesian analysis, information on the choice of priors and Markov chain Monte Carlo settings
☒	☐	For hierarchical and complex designs, identification of the appropriate level for tests and full reporting of outcomes
☐	☒	Estimates of effect sizes (e.g. Cohen's d, Pearson's r), indicating how they were calculated

Our web collection on statistics for biologists contains articles on many of the points above.

Software and code

Policy information about availability of computer code

Data collection	Single-cell RNA-Seq libraries from mouse tumors were prepared per the Single Cell 3′ v3.1 Reagent Kits User Guide (10x Genomics). Libraries were sequenced on a NovaSeq 600.<br>Immune fluorescence images were acquired using an Olympus SpinSR10 spinning disk confocal super-resolution microscope (Olympus, Japan).<br>Flow cytometry data was acquired on a CytoFLEX (Beckman).<br>The luciferase activity was measured with a Dual-Luciferase Assay (Promega; YEASEN) using a SpectraMax i3x Multi-Mode Microplate Reader (Molecular Devices, USA).<br>Quantitative real-time PCR data was acquired with the LightCycler ® 480 System.<br>Detailed information was provided in the Methods.
Data analysis	GraphPad Prism 8.0, FlowJo v.10.8, Cell Ranger v7.0.0, Seurat v.4.1.0, SAMtools v1.7, MarkDuplicates v.1.136, MACS2 v2.2.6, HOMER. A detailed description of Data analysis was provided in the Methods and the Supplemental Table 1.

For manuscripts utilizing custom algorithms or software that are central to the research but not yet described in published literature, software must be made available to editors and reviewers. We strongly encourage code deposition in a community repository (e.g. GitHub). See the Nature Portfolio guidelines for submitting code & software for further information.

Data

Policy information about availability of data

All manuscripts must include a data availability statement. This statement should provide the following information, where applicable:

- Accession codes, unique identifiers, or web links for publicly available datasets
- A description of any restrictions on data availability
- For clinical datasets or third party data, please ensure that the statement adheres to our policy

Data generated in this study have been deposited at NCBI-GEO (Super-Series GSE221938) and are publicly available now.
scRNA-seq from 60 patients with ESCC were retrieved from GSE160269.
RNA-seq datasets from patients with squamous cell carcinoma were retrieved from The Cancer Genome Atlas (TCGA).
RNA-seq datasets from SCC cell lines were collected from The Cancer Cell Line Encyclopedia (CCLE) and GSE106564, GSE88833, GSE4975.
ChIP-seq datesets were were either generated in house (GSE106563 and GSE148920) or re-analyzed from GSE78212, GSE46837.
Detailed information including hyperlink was provided in the Supplemental Table 1.

Research involving human participants, their data, or biological material

Policy information about studies with human participants or human data. See also policy information about sex, gender (identity/presentation), and sexual orientation and race, ethnicity and racism.

Reporting on sex and gender	Both male and female.
Reporting on race, ethnicity, or other socially relevant groupings	There is no race, ethnicity or other socially relevant groupings.
Population characteristics	Human ESCC and operative margin tissues were procured from surgical resection specimens. All of the patients received no treatment prior to surgery and signed separate informed consent forms for sample collection. Patients' age ranged from 45 to 71 years old, with a median age of 56.
Recruitment	Samples were collected from 1999 to 2020.
Ethics oversight	The study was approved by the Ethics Committee of the Cancer Institute (Hospital), Chinese Academy of Medical Sciences (CAMS) & Peking Union Medical College (PUMC) (No. 16-171/1250). All of the patients have signed separate informed consent forms for sample collection.

Note that full information on the approval of the study protocol must also be provided in the manuscript.

Field-specific reporting

Please select the one below that is the best fit for your research. If you are not sure, read the appropriate sections before making your selection.

☒ Life sciences ☐ Behavioural & social sciences ☐ Ecological, evolutionary & environmental sciences

For a reference copy of the document with all sections, see nature.com/documents/nr-reporting-summary-flat.pdf

Life sciences study design

All studies must disclose on these points even when the disclosure is negative.

Sample size	Sample sizes were not predetermined and were indicated in the figure legends. In general a minimun of 3 and up to 8 samples for each group were included. The number of mice per group (minimum 5) was decided based on previous experience (DOI: 10.1053/j.gastro.2020.06.050), common practice in the field, animal welfare guidelines and availability of animals, while minimizing the use of animals in accordance with animal care guidelines from the Cedars-Sinai Institutional Animal Care & Use Committee (CSMC IACUC) and the Hefei Institutes of Physical Science, Chinese Academy of Sciences.
Data exclusions	No data was excluded from the analysis.
Replication	Replication data was not performed for scRNA-seq due to the cost. Other attempts at replication were successful.<br>For in vitro experiments, a minimum of 3 independent replicates were performed at least in two cell lines.<br>In vivo syngeneic mouse models were repeated twice at least with 2 cell lines.<br>The number of biological replicates is as described in the figure legends.
Randomization	Age-matched mice were randomized into each group. For OT-I TCR (C57BL/6-Tg (TcraTcrb)1100Mjb/J) transgenic mice, mice with matched ages were used based on the genotypes.
Blinding	Phenotype, flow cytometry, RNA-Seq, ChIP-Seq and quantification analyses were performed with the investigators blinded to the genotype and/or condition.Tumor growth curves were not plotted until all the data points were collected. Experiments were performed blinded when possible.

Reporting for specific materials, systems and methods

We require information from authors about some types of materials, experimental systems and methods used in many studies. Here, indicate whether each material, system or method listed is relevant to your study. If you are not sure if a list item applies to your research, read the appropriate section before selecting a response.

Materials & experimental systems

n/a	Involved in the study
☐	☒ Antibodies
☐	☒ Eukaryotic cell lines
☒	☐ Palaeontology and archaeology
☐	☒ Animals and other organisms
☒	☐ Clinical data
☒	☐ Dual use research of concern
☒	☐ Plants

Methods

n/a	Involved in the study
☐	☒ ChIP-seq
☐	☒ Flow cytometry
☒	☐ MRI-based neuroimaging

Antibodies

Antibodies used

A detailed description was provided in the Supplemental Table 1 as below:
Rabbit Anti-p63 Antibody Abcam Cat# ab97865; 1:1000 for WB; 1:200 for IF
Rabbit Anti-p63-α Antibody Cell Signaling Technology Cat# 13109; clone D2K8X; 1:1000 for WB; 1:500 for IF; 1:100 for IP and ChIP
Rabbit Anti-p63 Antibody GeneTex Cat# GTX102425; clone N2C1; 1:1000 for WB; 1:400 for IF
Mouse Anti-CD8α Cell Signaling Technology Cat# 70306; clone C8/144B; 1:300 for IF
CD8 Monoclonal Antibody Thermo Fisher Scientific Cat# MA5-14548; clone SP16; 1:300 for IF
APC anti-mouse CD45 Antibody BioLegend Cat# 103112; clone 30-F11
Brilliant Violet 510™ anti-mouse CD3 Antibody BioLegend Cat# 100233; clone 17A2
FITC anti-mouse CD8a Antibody BioLegend Cat# 100706; clone 53-6.7
PE anti-mouse CD69 Antibody BioLegend Cat# 104507; clone H1.2F3
PerCP/Cyanine5.5 anti-human Granzyme B Recombinant BioLegend Cat# 372212; clone QA16A02
Alexa Fluor® 700 anti-mouse IFN-γ Antibody BioLegend Cat# 505823; clone XMG1.2
Purified Rat Anti-Mouse CD16/CD32 (Mouse BD Fc Block™) BD Biosciences Cat# 553142; Clone 2.4G2 (RUO)
InVivoMab anti-mouse PD-1 BioXcell Cat# BE0146; clone RMP1-14
InVivoMAb rat IgG2a isotype control BioXcell Cat# BE0089; clone 2A3
InVivoMab anti-mouse CD8α BioXcell Cat# BE0061; clone 2.43
InVivoMAb rat IgG2b isotype control BioXcell Cat# BE0090; clone LTF-2
InVivoMAb anti-mouse IFNγ BioXcell Cat# BE0055; clone XMG1.2
InVivoMAb rat IgG1 isotype control BioXcell Cat# BE0088; clone HRPN
Phospho-STAT1 (Ser727) Polyclonal Antibody Invitrogen Cat# 44-382G; 1: 500 for WB; 1:200 for IF; 1: 50 for IP
STAT1 [p Tyr701] Antibody Novus Biologicals Cat# AF2894-SP; 1:1000 for WB; 1: 50 for IP;
Stat1 Antibody Cell Signaling Technology Cat# 9172; 1:1000 for WB; 1: 50 for IP
Rabbit Anti-GAPDH (14C10) Cell Signaling Technology Cat# 2118; 1:2000 for WB
Peroxidase AffiniPure Goat Anti-Rabbit IgG (H+L) Jackson ImmunoResearch Laboratories, Inc Cat# 111-035-045; 1:10000 for WB
Peroxidase AffiniPure Goat Anti-Mouse IgG (H+L) Jackson ImmunoResearch Laboratories, Inc Cat# 115-035-003; 1:10000 for WB
Mouse anti-Human BATF2 / SARI Antibody LSBio Cat# LS C541060; 1:1000 for WB
Beta-2-Microglobulin Polyclonal antibody Proteintech Cat# 13511-1-AP; 1:4000 for WB
PD-L1/CD274 Monoclonal antibody Proteintech Cat# 66248-1-Ig; 1:2000 for WB
IFNGR1 Rabbit Polyclonal Antibody Novus Biologicals Cat# AF7176; 1:500 for WB
IFNGR2 Polyclonal antibody Proteintech Cat# 10266-1-AP; 1:1000 for WB
IRF1 Polyclonal antibody Proteintech Cat# 11335-1-AP; 1:500 for WB
MHC class I (HLA-A/B) Rabbit mAb ABclonal Cat# A8754; 1:1000 for WB
Rabbit secondary antibody Abcepta Cat# ASP1615; 1:10000 for WB
Mouse secondary antibody Abcepta Cat# ASP1613; 1:10000 for WB
Fixable Viability Stain 450 BD Biosciences Cat# 562247
Donkey anti-Mouse IgG (H+L) Highly Cross-Adsorbed Secondary Antibody,
Alexa FluorTM 488 Invitrogen Cat# A-21202; 1:5000 for IF
Donkey anti-Mouse IgG (H+L) Highly Cross-Adsorbed Secondary Antibody, Alexa FluorTM 594 Invitrogen Cat# A-21203; 1:1000 for IF
Donkey anti-Rabbit IgG (H+L) Highly Cross-Adsorbed Secondary Antibody, Alexa FluorTM 488 Invitrogen Cat# A-21206; 1:1000 for IF
Donkey anti-Rabbit IgG (H+L) Highly Cross-Adsorbed Secondary Antibody, Alexa FluorTM 594 Invitrogen Cat# A-21207; 1:3000 for IF
Rabbit secondary antibody GE Healthcare Cat# NXA934; 1:10000 for WB
Mouse secondary antibody GE Healthcare Cat# NXA931; 1:10000 for WB

Validation

All antibodies are from commercially available sources and have been validated by the manufacturers with supporting data and publications found on the manufacturers' websites.
Flow cytometry antibodies in this study from BioLegend and BD Biosciences have been widely used in the last years and have been

validated for manufactures.
Western blot, co-immunoprecipitation and immunofluorescence antibodies were validated for manufactures.
In vivo monoclonal antibodies have been widely used in the last years for scientific community.

Eukaryotic cell lines

Policy information about cell lines and Sex and Gender in Research

Cell line source(s)	TE5 and TT cell lines were kindly provided by Dr. Koji Kono (Cancer Science Institute of Singapore, Singapore).<br>AKR and HNM007 cell lines were a gift from Dr. Anil K. Rustgi (Columbia University Irving Medical Center, USA).<br>MOC1, MOC22 were from the laboratory of Dr. Ravindra Uppaluri (Dana-Farber Cancer Institute, USA).
Authentication	All the cell lines were authenticated through short tandem repeat (STR) analysis before using in experiments.
Mycoplasma contamination	Cell lines were tested for mycoplasma contamination using the Mycoplasma PCR Detection Kit. Cells with mycoplasma contamination were discarded immediately.
Commonly misidentified lines (See ICLAC register)	No commonly misidentified lines were used in the study.

Animals and other research organisms

Policy information about studies involving animals; ARRIVE guidelines recommended for reporting animal research, and Sex and Gender in Research

Laboratory animals	C57BL/6J, C57BL/6-Tg (TcraTcrb)1100Mjb/J and C57BL/6JGpt were used in this study.<br>All the mice were housed in a specific pathogen-free facility: in a 12h light/dark cycle (lights on at 08:00h), ambient temperature of 20-24°C with 40-60% humidity.<br>OT-I TCR transgenic mice were authenticated with PCR before using in experiments.<br>Syngeneic mouse models were conducted in mice between 6-10 weeks-old.<br>Detailed descriptions were provided in the Methods and Supplemental Table 1.
Wild animals	No wild animals were used in the study.
Reporting on sex	Male mice were used for syngeneic murine SCC models because SCC is more common in men than women. However, it has been demonstrated that the gender of recipient mice did not have a major impact on xenograft tumor development, therefore, mice of both gender are appropriate for use to study the xenograft tumor development.
Field-collected samples	No field-collected samples were used in the study
Ethics oversight	Animal studies were respectively approved and performed according to the ethical regulations of Cedars-Sinai Institutional Animal Care & Use Committee (CSMC IACUC) and the animal care regulations of Hefei Institutes of Physical Science, Chinese Academy of Sciences.

Note that full information on the approval of the study protocol must also be provided in the manuscript.

Plants

Seed stocks	This study did not involve seed stocks.
Novel plant genotypes	This study did not invlolve novel plant genotypts.
Authentication	This study did not invlolve novel plant genotypts.

ChIP-seq

Data deposition

- [x] Confirm that both raw and final processed data have been deposited in a public database such as GEO.
- [x] Confirm that you have deposited or provided access to graph files (e.g. BED files) for the called peaks.

Data access links<br>*May remain private before publication.*	ChIP-seq datesets were were either generated in house (GSE106563 and GSE148920) or re-analyzed from GSE78212, GSE46837.
Files in database submission	Our in house data (GSE106563 and GSE148920) and data from GSE78212 and GSE46837 are publicly available now.
Genome browser session (e.g. UCSC)	Not applicable

Methodology

Replicates	A detailed description was provided in Methods.
Sequencing depth	A detailed description was provided in Methods.
Antibodies	A detailed description was provided in Methods.
Peak calling parameters	A detailed description was provided in Methods.
Data quality	A detailed description was provided in Methods.
Software	A detailed description was provided in Methods.

Flow Cytometry

Plots

Confirm that:

- [x] The axis labels state the marker and fluorochrome used (e.g. CD4-FITC).
- [x] The axis scales are clearly visible. Include numbers along axes only for bottom left plot of group (a 'group' is an analysis of identical markers).
- [x] All plots are contour plots with outliers or pseudocolor plots.
- [x] A numerical value for number of cells or percentage (with statistics) is provided.

Methodology

Sample preparation	For immune profiling assays, single cell suspension of tumors were obtained by manual dissociation with rapid physical grinding and digestion in 5 mg/mL collagenase IV and 0.25% Trypsin-EDTA supplemented with 10 U/mL DNase I. Immune cells were enriched using CD45 MicroBeads and blocked with Fc Block™ antibody. Live cells were determined with Fixable Viability Stain 450 then stained with appropriate antibodies. To quantify intracellular markers, fixation and permeabilization with True-Nuclear™ Transcription Factor Buffer Set were performed before incubation of antibodies.<br>For T cell-mediated tumor cell killing assays, CD8+ T cells were obtained from the spleen of OT-I TCR mice and activated with OVA257-264 peptide. Successful enrichment and activation of CD8+ T cells were confirmed by flow cytometry using antibodies against CD45, CD3, CD8, and CD69.
Instrument	Flow cytometry was performed on a CytoFLEX (Beckman)
Software	Data were analyzed using FlowJo v10.8.1 software.
Cell population abundance	Different cell populations were measured by flow cytometry in mice samples.
Gating strategy	UltraComp eBeads were used for caculation of compensation with fluorochromes excited by different lasers. Single cells were first gated with FSC-A and FSC-H. Live cells were determined with Fixable Viability Stain 450. Total immune cells were identified by CD45 staining. Subsequent gating was conducted to selected targeted populations.

- [x] Tick this box to confirm that a figure exemplifying the gating strategy is provided in the Supplementary Information.
